# The Pervasive Effects of an Antibiotic on the Human Gut Microbiota, as Revealed by Deep 16S rRNA Sequencing

**DOI:** 10.1371/journal.pbio.0060280

**Published:** 2008-11-18

**Authors:** Les Dethlefsen, Sue Huse, Mitchell L Sogin, David A Relman

**Affiliations:** 1 Department of Microbiology and Immunology, Stanford University, Stanford, California, United States of America; 2 Department of Medicine, Stanford University, Stanford, California, United States of America; 3 Josephine Bay Paul Center for Comparative Molecular Biology and Evolution, Marine Biological Laboratory, Woods Hole, Massachusetts, United States of America; 4 Veterans Affairs Palo Alto Health Care System, Palo Alto, California, United States of America; University of California, Davis, United States of America

## Abstract

The human intestinal microbiota is essential to the health of the host and plays a role in nutrition, development, metabolism, pathogen resistance, and regulation of immune responses. Antibiotics may disrupt these coevolved interactions, leading to acute or chronic disease in some individuals. Our understanding of antibiotic-associated disturbance of the microbiota has been limited by the poor sensitivity, inadequate resolution, and significant cost of current research methods. The use of pyrosequencing technology to generate large numbers of 16S rDNA sequence tags circumvents these limitations and has been shown to reveal previously unexplored aspects of the “rare biosphere.” We investigated the distal gut bacterial communities of three healthy humans before and after treatment with ciprofloxacin, obtaining more than 7,000 full-length rRNA sequences and over 900,000 pyrosequencing reads from two hypervariable regions of the rRNA gene. A companion paper in *PLoS Genetics* (see Huse et al., doi: 10.1371/journal.pgen.1000255) shows that the taxonomic information obtained with these methods is concordant. Pyrosequencing of the V6 and V3 variable regions identified 3,300–5,700 taxa that collectively accounted for over 99% of the variable region sequence tags that could be obtained from these samples. Ciprofloxacin treatment influenced the abundance of about a third of the bacterial taxa in the gut, decreasing the taxonomic richness, diversity, and evenness of the community. However, the magnitude of this effect varied among individuals, and some taxa showed interindividual variation in the response to ciprofloxacin. While differences of community composition between individuals were the largest source of variability between samples, we found that two unrelated individuals shared a surprising degree of community similarity. In all three individuals, the taxonomic composition of the community closely resembled its pretreatment state by 4 weeks after the end of treatment, but several taxa failed to recover within 6 months. These pervasive effects of ciprofloxacin on community composition contrast with the reports by participants of normal intestinal function and with prior assumptions of only modest effects of ciprofloxacin on the intestinal microbiota. These observations support the hypothesis of functional redundancy in the human gut microbiota. The rapid return to the pretreatment community composition is indicative of factors promoting community resilience, the nature of which deserves future investigation.

## Author Summary

The intestinal microbiota is essential to human health, with effects on nutrition, metabolism, pathogen resistance, and other processes. Antibiotics may disrupt these interactions and cause acute disease, as well as contribute to chronic health problems, although technical challenges have hampered research on this front. Several recent studies have characterized uncultured and complex microbial communities by applying a new, massively parallel technology to obtain hundreds of thousands of sequences of a specific variable region within the small subunit rRNA gene. These shorter sequences provide an indication of diversity. We used this technique to track changes in the intestinal microbiota of three healthy humans before and after treatment with the antibiotic ciprofloxacin, with high sensitivity and resolution, and without sacrificing breadth of coverage. Consistent with previous results, we found that the microbiota of these individuals was similar at the genus level, but interindividual differences were evident at finer scales. Ciprofloxacin reduced the diversity of the intestinal microbiota, with significant effects on about one-third of the bacterial taxa. Despite this pervasive disturbance, the membership of the communities had largely returned to the pretreatment state within 4 weeks.

## Abstract 3

The most complete survey to date of bacterial diversity in the human gut shows extensive but temporary changes in the microbial community following ciprofloxacin treatment.

## Introduction

Specialized microbial communities inhabit the skin, mucosal surfaces, and gastrointestinal tract of humans (and other vertebrates) from birth until death, with by far the largest populations in the colon [1,2]. Humans rely on their native microbiota for nutrition and resistance to colonization by pathogens [3–6]; furthermore, recent discoveries have shown that symbiotic microbes make essential contributions to the development, metabolism, and immune response of the host [7–10]. Co-evolved, beneficial, human–microbe interactions can be altered by many aspects of a modern lifestyle, including urbanization, global travel, and dietary changes [1], but in particular by antibiotics [11]. The acute effects of antibiotic treatment on the native gut microbiota range from self-limiting “functional” diarrhea to life-threatening pseudomembranous colitis [12,13]. The long-term consequences of such perturbations for the human–microbial symbiosis are more difficult to discern, but chronic conditions such as asthma and atopic disease have been associated with childhood antibiotic use and an altered intestinal microbiota [14–16]. Because many chemical transformations in the gut are mediated by specific microbial populations [17], with implications for cancer [18,19] and obesity [20,21], among other conditions [22], changes in the composition of the gut microbiota could have important but undiscovered health effects. An approximate return to pretreatment conditions often (but not always) occurs within days or weeks after cessation of antibiotic treatment, as assessed by subjective judgments of bowel function and characterizations of overall community composition using techniques with low phylogenetic resolution [23–25]. However, the effects of a single course of antibiotics on specific microbial populations in vivo can persist for years [26–28]. Overall, the duration and extent of antibiotic-induced disturbance throughout the intestinal microbiota remains poorly characterized, particularly at the species and strain level where the diversity of the community is greatest [2,29], and we lack the information to compare these disturbances to normal temporal variation in community composition.

The diversity and abundance of the human microbiota, and of the gut community in particular, poses a challenge for researchers investigating changes in community composition over time. The laborious cultivation-based techniques that were the mainstay of microbiology for a century have revealed only a minority of the species that inhabit the human colon [30,31]. Over the past decade, cultivation-independent molecular techniques, particularly those based on the small subunit ribosomal RNA (16S rRNA) gene, have given us a broader and less biased view of the gut microbiota [32,33]. Full-length 16S rRNA sequences offer the highest possible degree of taxonomic resolution using this gene, but the cost of dideoxy Sanger sequencing limits our ability to survey the less-abundant members of this diverse community. Studies comparing changes in the gut microbiota over time or between treatments have often used alternative molecular techniques that are more rapid and less expensive than sequencing, but that offer lower taxonomic resolution and reveal only the more abundant members of the community [23–25,34–37]. These approaches can be focused more narrowly on particular taxonomic groups, which facilitates the investigation of less-abundant taxa, but at the expense of a broader view of community composition that might reveal important microbial interactions.

Pyrosequencing ameliorates some of these constraints by generating a much larger amount of genetic sequence data at a lower cost [38]. Both with this approach and with the established approach of clone library sequencing, DNA is extracted from a sample, and PCR primers complementary to conserved regions of the 16S rRNA are used to amplify the intervening variable sequence. The diversity and relative abundance of 16S rRNA sequence variants in the pool of amplicons is analyzed as a proxy for the diversity and relative abundance of the microbial populations in the sample. Because the gene sequence-based recognition of uncultivated microbial populations is not equivalent to traditional taxonomic classification, terms such as “species” or “strain” are not appropriate. Instead, the populations inferred to exist on the basis of sequence data are referred to as operational taxonomic units (OTUs), which can be defined in various ways and at different levels of resolution.

Both the clone library and pyrosequencing approaches can be affected by biased PCR amplification of microbial populations in the sample, although the problem is reduced for the shorter pyrosequencing amplicons [39]. The pyrosequencing approach also avoids the cloning bias of the earlier technique by attaching amplicons individually to beads and physically separating the beads into picoliter-scale wells on a specialized plate. Less phylogenetic information is available from a single pyrosequencing read (at most, ∼230 informative bases with recent technology) than from near full-length 16S rRNA gene sequence (∼1,400 bases with commonly used primers), but reads that span particular variable regions of the gene are highly informative [40,41]. On the other hand, well over 200,000 short 16S rRNA sequence reads, which we refer to as tags, can be obtained in a single run of the Genome Sequencer FLX System. In comparison, two runs of a state-of-the-art capillary Sanger sequencer are required to obtain at most 384 full-length 16S rRNA sequences.

Sogin and his co-workers have demonstrated the power of pyrosequencing by amplifying the V6 variable region of 16S rRNA from marine deep water and hydrothermal vent samples, analyzing over 900,000 bacterial and archaeal tags from the vent system and revealing greater taxonomic richness (over 20,000 OTUs observed at 3% sequence divergence) than has previously been reported for any microbial habitat [39,42,43]. Roesch et al. followed a similar strategy using the V9 region to analyze North American soil samples [40,44], and a group led by Knight have focused on a longer tag that includes the V2 region in macaque gut samples and in a mixture of five diverse microbial habitats [45,46]. Computer simulations by Liu et al. [43], Wang et al. [41], and Sundquist et al. [47] have indicated that pyrosequencing tags from different regions of the gene will vary in their utility for the distinct tasks of revealing microbial diversity and performing taxonomic classification, both of which contribute to making informative comparisons between complex microbial communities. As with the established approach of generating data from clone libraries, the actual performance of tag pyrosequencing for the goals of a particular study will depend on the region of the 16S rRNA gene analyzed, the PCR primers used, and the composition of the microbial communities in the samples.

In the present study, we used pyrosequencing tags spanning the V6 and V3 regions as well as full-length 16S rRNA sequences to analyze the bacterial community composition in a time series of stool samples obtained from three healthy adults before, during, and after a short course of the antibiotic ciprofloxacin (Cp). A companion paper [48] reports a high degree of similarity in the community composition inferred from the two variable regions and from full-length sequences. Here we describe the diversity of human intestinal bacteria more completely and with more precision than has previously been possible. Cp has an extensive and individualized effect on the composition of these communities, but most members of the community returned to their pretreatment abundance within weeks.

## Methods

### Participants, Treatment, and Sampling

Healthy adults who had not taken any antibiotics within the previous year were recruited to donate stool samples before, during, and after a short course of ciprofloxacin (Cp) (500 mg twice a day for 5 d), typical of the treatment prescribed, e.g., for an uncomplicated urinary tract infection. Samples of approximately 5 g were collected in sterile plastic containers by the participants themselves and immediately stored in home freezers until brought to the laboratory (within several days) for storage at −80 °C. From a larger number of samples collected, we chose five time points spanning an 8-mo interval from each of three participants for analyses using full-length 16S rRNA clone libraries and pyrosequencing of the V6 variable region of 16S rRNA. An additional three samples from one of the participants were analyzed along with the original 15 samples using V3 tag pyrosequencing. Participant characteristics and the sampling and analysis regime are shown in Table 1. For each participant, samples 1 and 2 (1, 2a, 2b, and 2c for individual A) were collected prior to Cp treatment, sample 3 (3a and 3b for individual A) was the Cp-associated sample taken during or immediately after treatment, and samples 4 and 5 were more distant post-treatment samples. Informed consent was obtained from participants, and the study protocol was approved by the Administrative Panel for Medical Research on Human Subjects (Institutional Review Board) of Stanford University.

**Table 1:**
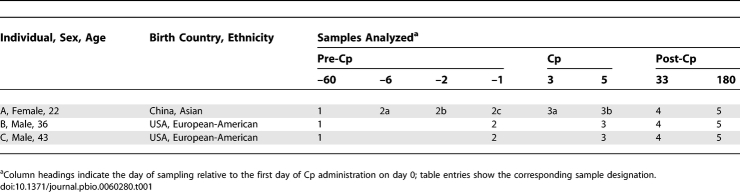
Features of Participants and Samples

### DNA Extraction

A subsample of approximately 200 mg of frozen stool was added to a 2.0-ml screwcap vial containing glass beads of 1 mm, 0.5 mm, and 0.1 mm diameter (BioSpec Products), and kept on ice until the addition of 1.4-ml ASL buffer from the QIAamp DNA Stool Mini Kit (Qiagen). Samples were immediately subjected to beadbeating (45 s, setting 4) using a FastPrep machine (Bio 101) prior to the initial incubation for heat and chemical lysis at 95 °C for 7 min. Subsequent steps of the DNA extraction followed the QIAamp kit protocol. DNA from two separate extractions of each sample were pooled for molecular analysis. Extraction controls lacking fecal material but otherwise treated identically were carried through the entire procedure including PCR amplification (no bands visible) and attempted cloning of PCR amplicons, which was unsuccessful.

### Full-Length 16S rRNA Sequencing

To amplify near full-length 16S rRNA sequences, 2 ml of extracted DNA served as the template in 20-μl reactions containing 1 unit AmpliTaq polymerase (Applied Biosystems), 1x Buffer II, 1.5mM MgCl_2_, 20mM tetramethylammonium chloride, 5% DMSO, 0.02% Triton X-100, 100 nM of each dNTP, and 20 nM each of forward and reverse primers (Integrated DNA Technologies). The forward primer was a mixture of 90% bacterial primer 8F (5′-AGAGTTTGATCMTGGCTCAG-3′) and 10% 8F-Bif targeting *Bifidobacteria* (5′-AGGGTTCGATTCTGGCTCAG-3′); it was paired with the three domain reverse primer 1391R (5′-GACGGGCGGTGTGTRCA-3′). Thermocycling involved a 5-min denaturation at 95 °C followed by 15–22 cycles of 94 °C (30 s), 55 °C (30 s), and 72 °C (90 s), with a final extension at 72 °C for 8 min. The fewest possible cycles were used to produce a faint band of the expected size under UV illumination after electrophoresis of 5-μl reaction product in a 1% agarose minigel containing 1 mM ethidium bromide. Two 4-cycle, 50-μl reconditioning PCR reactions were performed per sample to eliminate heteroduplexes [49], with 5-μl aliquots of the initial PCR product mixture as the template and other PCR conditions unchanged. Products of the two reconditioning PCR reactions per sample were combined, purified using QIAquick PCR purification columns (Qiagen), and sent to the J. Craig Venter Institute (Rockville, MD) for cloning and automated bidirectional dideoxy sequencing.

Forward and reverse sequencing reads were assembled using the Phred/Phrap/Consed suite of programs with default parameters [50–52]; assembled sequences were aligned via the NAST algorithm [53] at the Greengenes Website (http://greengenes.lbl.gov/) [54]. Aligned sequences were checked for chimeras using version 2 of the Greengenes implementation of Bellerophon, with 99% similarity to a trusted sequence over 1,250 nucleotides as the threshold to bypass checking, and rejecting both putative and subthreshold chimeras regardless of the divergence ratio. Of 10,062 assembled sequences, 203 did not result in a NAST alignment that included both the V3 and V6 regions, and 2,651 were excluded as possible chimeras, leaving 7,208 full-length sequences for analysis. Using the less-stringent default settings in Bellerophon to identify chimeras (divergence ratio > 1.1) would have excluded 766 fewer sequences from the analysis, but the exclusion of these sequences did not significantly affect the inferred taxonomic composition of the community (Text S1). The taxonomic affiliation of full-length sequences was determined using the Classifier tool of the RDP, with an 80% bootstrap threshold [41].

The NAST-aligned full-length sequences were imported into ARB [55] and a genetic distance matrix calculated with the Olsen distance correction using the Hugenholtz version of the Lane mask (included with Greengenes ARB database) to exclude regions of questionable alignment; 1,241 columns were retained. The distance matrix was used with a 0.01 genetic distance, furthest-neighbor threshold in DOTUR version 1.53 [56] to designate 1,295 operational taxonomic units (OTU_0.01_). The full-length sequences have been deposited in GenBank (http://www.ncbi.nlm.nih.gov/Genbank/; accession numbers: EU761594–EU768801).

### 16S rRNA Tag Pyrosequencing

Amplicon libraries for 16S rRNA V6 region pyrosequencing were generated as recommended by 454 Life Sciences, using the same DNA extracts as for full-length sequencing, with 50 ng of template DNA (determined by a NanoDrop 1000 spectrophotometer, NanoDrop Technologies) in 50-μl, 30-cycle reactions. The PCR reagents and thermocycling parameters were those suggested in the protocol, except that the annealing temperature was reduced from 57 °C to 55 °C. Each PAGE-purified fusion primer (Integrated DNA Technologies) consisted of the 454 platform A or B linker/primer sequence, a trinucleotide key (or barcode) that was unique for each sample, and sequence complementary to a conserved region flanking the V6 variable region of the 16S rRNA gene. The forward primer was an equimolar mixture of two nondegenerate oligonucleotides 5'-*Bxxx*CAACGCGAAGAACCTTACC-3′ and 5'-*Bxxx*ATACGCGAGGAACCTTACC-3′, where *B* represents the B linker (5′-GCCTTGCCAGCCCGCTCAG-3′), *xxx* represents the sample identification key, and the remaining nucleotides correspond to positions 967–985 of the 16S rRNA gene (Escherichia coli numbering). The degenerate reverse primer was 5'-*Axxx*CGACARCCATGCASCACCT-3′, including the A linker (5′-GCCTCCCTCGCGCCATCAG-3′), sample key, and nucleotides corresponding to E. coli positions 1,064–1,046. Two or three separate amplification reactions for each sample were pooled and purified as recommended using Ampure beads (Agencourt).

The amplicon length and concentration in the libraries was estimated using the BioAnalyzer microfluidics device (Agilent), and an equimolar mix of all 15 V6 amplicon libraries was used to prepare both A-linked and B-linked pyrosequencing beads (i.e., for bidirectional reads of the amplicons) via emulsion PCR using the recommended kit and protocol (454 Life Sciences). An equal mixture of A-linked and B-linked beads was loaded onto both regions of a Picotiter plate (estimated 825,000 beads/region). Pyrosequencing on the Genome Sequencer FLX System (Roche) at the Stanford Genome Technology Center resulted in 490,881 reads which passed the length and quality criteria of the machine software.

Preparation of amplicon libraries for the V3 region used the same DNA extracts as for the full-length and V6 sequences, except that new DNA extracts were prepared for samples 2c and 3b from individual A, and additional samples 2a, 2b, and 3a from individual A were included, as described above and in Table 1. V3 amplicon libraries were prepared as for the V6 region, using a single 50-μl PCR reaction per sample and with the following PAGE-purified primers (Integrated DNA Technologies): 5'-*Bxxxxx*ACTCCTACGGGAGGCAGCAG-3′ (B linker, pentamer sample identification key, forward primer at E. coli positions 338–357) and 5'-*Axxxxx*TTACCGCGGCTGCTGGCAC-3′ (A linker, pentamer key, reverse primer at E. coli positions 533–515). Preparation of A-linked and B-linked beads and pyrosequencing were carried out as described above, with an estimated 900,000 beads loaded in each region of the Picotiter plate, which resulted in 593,088 pyrosequencing reads passing machine quality filters.

Pyrosequencing reads of both the V6 and V3 regions were passed through additional quality filters to reduce the overall error rate [43]. Any reads containing one or more ambiguous nucleotides and reads shorter than 50 nucleotides were discarded. The expected sample keys and primer sequences were trimmed from the proximal and distal ends of the reads, and those lacking an expected sample key and primer sequence at either end were examined further. In many of these cases, a rare variant of the expected sequence was identified and trimmed from the read. The origin of at least some of these rare primer variants as oligonucleotide synthesis errors was suggested by the frequent appearance of several copies of a particular rare variant in a single sample, while no examples of that variant appeared in any other sample. Because unique primers (differing in the sample key and synthesized separately) were used to generate the amplicon libraries from each sample, a synthesis error would be likely to affect only a single sample. Reads lacking a recognizable primer sequence (including rare variants) at either end, reads covering only a portion of the variable region, and reads that could not be unambiguously assigned to a sample were discarded; in addition, 18 reads from the V3 region with identity or near identity to eukaryotic rRNA sequences were discarded. The resulting datasets contained 441,894 V6 tags and 490,699 V3 tags, which were the basis of all subsequent analyses.

### Reference Databases and OTU Definition for Tag Pyrosequencing

The V6 and V3 reference databases (V6 RefDB, V3 RefDB) are composed of publically available, high quality, full-length 16S rRNA sequences from Silva release 92 (http://www.arb-silva.de/)[57] with taxonomic classifications obtained from the RDP Classifier [41] (minimum 80% bootstrap score) as described in [48]. The V6 region is defined as E. coli positions 986-1045 corresponding to the sequence between primers 967F and 1046R. The V3 region is defined as *E. coli* positions 358–514, corresponding to the sequence between primers 338F and 515R.

Pyrosequencing tags were assigned the taxonomic classification of the most similar reference sequence or sequences in the V6RefDB or V3RefDB, based on a multiple sequence alignment. In cases where a tag was equidistant to multiple reference sequences, the tag was classified to the level of the most resolved taxon shared by at least two-thirds of the reference sequences nearest that tag. For example, a tag with exact matches to multiple reference sequences would be resolved to the genus level only if at least two-thirds of the sequences containing that tag were classified in the same genus with an 80% bootstrap score by the RDP.

**Figure 1:**
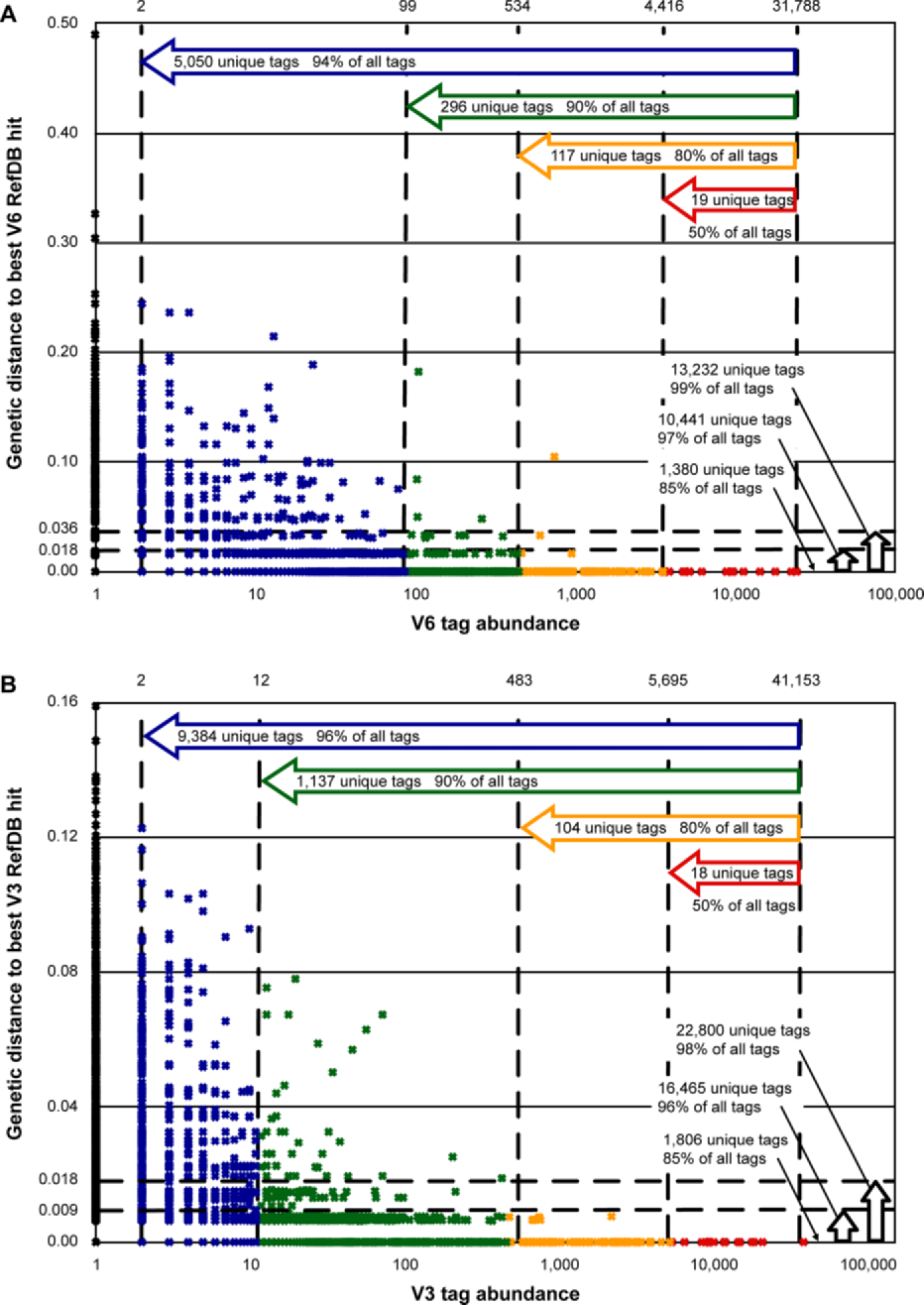
Genetic Distances between Tags and Reference Sequences The genetic distances between each unique V6 tag (A) and V3 tag (B) and its best hit (or hits) in the appropriate reference database is shown on the vertical axis (see Methods for details); the abundance of the tag is shown on a logarithmic scale on the horizontal axis. Horizontal arrows indicate cumulative totals of tags from most to least abundant. Discrete horizontal rows of symbols near the *x*-axis correspond to an integer number of nucleotide changes relative to a reference sequence; vertical arrows indicate cumulative tag totals for up to 0, 1, or 2 changes. Abundant tags are likely to be identical to sequences in the reference databases; rare tags display a range of distances.

A reference sequence-based operational taxonomic unit (refOTU) was defined as containing all query tags sharing the same RefDB sequence (or group of sequences) as their nearest neighbor. The vast majority of human gut pyrosequencing tags obtained in the current study (unlike marine tags [39]) have an exact or near match in the corresponding RefDB (Figure 1), which simplifies the classification of tags and the definition of refOTUs for this habitat. In this study, we will refer to a specific refOTU as V3*_Taxon* refOTU_X, where *Taxon* is the most-resolved taxon name assigned to the refOTU, and X is the rank order of the refOTU within *Taxon* based on the total normalized abundance data from this study.

### Statistical Calculations

The comparison of nearest database distance to tag abundance, the rank abundance plots, the rarefaction curves, and the diversity summary statistics were calculated using actual tag counts without normalizing the number of tags obtained per sample. Rarefaction curves for full-length sequence OTU_0.01_s were calculated on a per-tag basis using DOTUR version 1.53 [58]; rarefaction curves for variable region refOTUs were calculated per sample using EstimateS version 7.5 [59]. The Shannon diversity index *H* = –Σ *p_i_* ln(*p_i_*) and Shannon equitability index *E_H_* = *H*/ln(*S*) (where *p_i_* is the proportion of the *i*th OTU and *S* is the total number of OTUs) were calculated using spreadsheet software. The diversity summary statistics for Cp-associated samples were compared to the lower tail of values for other samples, assuming that summary statistics would be distributed normally when calculated for samples drawn repeatedly from an unchanging community.

Comparisons of V3 refOTU abundance between samples were made after correcting for the unequal number of tags obtained from each sample. All tag abundance values within a sample were multiplied by the required factor so that total normalized tag abundance for all samples was 43,405, which was the number of V3 tags obtained from sample C5, the maximum for any sample. Normalization factors for the other samples ranged from 1.072 to 2.087. Principal component analysis of normalized refOTU abundance used the *prcomp* function of the *stats* package of the R statistical language, version 2.2.1 (http://www.r-project.org/). Statistical assessment of variation in the abundance of individual refOTUs between participants or with respect to Cp was conducted with Edge software, version 1.1.291 [59], applying both a 95% confidence level for individual taxa to declare significance, and a 10% false discovery rate (FDR) criterion for all taxa declared significant in a given comparison (additional details in Text S1).

## Results

### Number of Tags and Sequences

From 15 stool samples (five each from individuals A, B, and C), we obtained 7,208 near full-length 16S rRNA sequences by traditional PCR amplification, cloning, and capillary dideoxy sequencing. From the same set of 15 samples, we obtained 441,894 V6 pyrosequencing tags (average length 59), and from the same samples plus three additional samples from individual A (18 samples total), we obtained 490,699 V3 pyrosequencing tags (average length 145) (Table 2). The number of unique tags or sequences revealed by each method followed the same rank order as the total number of tags or sequences (V3 > V6 > full-length). However, the numerical disparity between pyrosequencing and capillary sequencing was smaller for unique tags or sequences than for all tags or sequences, reflecting the competing trends of higher throughput for tag pyrosequencing but greater resolution for full-length sequences. The V6 and V3 tags were grouped into 3,316 and 5,671 refOTUs, respectively, via comparison to reference databases (see Methods); 1,295 OTUs were identified among full-length sequences using a 1% genetic distance threshold after masking sequence positions of uncertain alignment (designated as OTU_0.01_). The number of unique genera or other most-resolved taxa above the genus level (see Methods) identified among sequences or tags was much lower, ranging from 56–130, confirming conclusions based on earlier molecular studies that the diversity of human colonic bacteria is concentrated at the species and strain level [2,29].

**Table 2:**
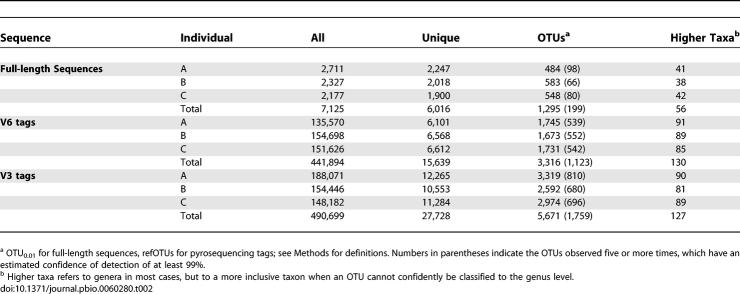
Numbers of Sequences, OTUs, and Taxa per Participant (A, B, and C)

The abundant pyrosequencing tags derived from these three participants were much more likely than were the rare tags to have been seen before, i.e., to correspond perfectly to one or more of the full-length 16S rRNA sequences already available in the public databases. Even though only 9% of the unique V6 tags and 7% of the unique V3 tags had exact matches in the reference databases, these represented 90% of all V6 tags and 85% of all V3 tags, because nearly all the abundant tags had exact matches. Of the 100 most abundant V6 and V3 tags (representing 75% and 80% of all tags, respectively), 97 V6 tags and 94 V3 tags had perfect matches in the reference databases. The abundant gut bacteria from these participants were less well represented by cultivated strains; only about half of the 100 most abundant tags (51 V6 tags and 55 V3 tags) had perfect matches to 16S rRNA sequences derived from bacterial strains in culture (Dataset S1).

### Pyrosequencing Errors and Rare, Genuine Tags

The recently reported average accuracy per base of better than 99.75% for pyrosequencing of the V6 region [43] (after applying additional filters to eliminate error-prone reads) is lower than that of capillary dideoxy sequencing, although considerably better than the 96% accuracy initially reported [38]. Undoubtedly, some fraction of the unique tags in our data arose from pyrosequencing errors, but individual error products were rare relative to the frequency of the correctly sequenced tag from which they were derived. The most abundant V6 and V3 tags in our data (exactly matching Bacteroides dorei sequences) were observed 31,788 and 41,153 times, respectively, across all three participants. Assuming a uniform error rate per base of 0.25% over 59 or 145 nucleotides, most tags are expected to be error-free, and most error-containing tags are expected to have only a single error. There were 177 unique V6 tags in our dataset that differed from the B. dorei V6 tag at a single nucleotide position and that lacked an exact match to any sequence in the reference database. The most abundant of these potential error products occurred only 60 times, 0.19% the frequency of the error-free B. dorei V6 tag. Similarly, of 270 unique V3 tags differing from the B. dorei V3 tag at only one position and lacking a perfect database match, the most abundant occurred 367 times. However, we consider this to be a rare, genuine tag, because the distribution of this tag among samples differed significantly from that of the B. dorei tag, in contrast to the pattern expected for a pyrosequencing error product (following paragraph; also Text S1 and Dataset S2). The most abundant potential V3 error product derived from the B. dorei sequence occurred 226 times, 0.55% the frequency of the error-free tag.

Sogin et al. suggested that using a reference database of public full-length 16S rRNA sequences to define taxa (i.e., refOTUs) for pyrosequencing tags would result in a conservative estimate of taxonomic richness [39]. This procedure will also minimize the influence of pyrosequencing errors on comparisons of community composition, since such errors could never result in the definition of a novel (but artificial) taxon. Instead, most pyrosequencing error products will be counted appropriately as a member of the refOTU containing the nearly identical error-free tag from which it was derived. However, this OTU definition inherently limits the detectable taxonomic diversity to the sequences already present in the public databases, and it obscures genuine biodiversity whenever tags derived from distinct organisms are grouped into a single refOTU. We investigated this diversity-masking effect for the most abundant V6 and V3 taxa by calculating, for each sample separately, the abundance ratios of individual rare tags to the most common tag belonging to the same refOTU (Text S1 and Dataset S2). For a given rare tag, significant variation in this ratio across samples is not expected if the rare tag is a pyrosequencing error product, because the likelihood of a particular error is independent of the origin of the tag. On this basis, four of the 42 rare V6 tags and 33 of the 87 rare V3 tags that we investigated were not likely to be pyrosequencing error products derived from the most common tag in the same refOTU (G test, *p* < 0.05). Even though this approach cannot differentiate all error-free, rare tags from pyrosequencing error products, it is clear that analyzing pyrosequencing tags on the basis of refOTUs will obscure some genuine biodiversity. Nonetheless, we chose to accept this cost in order to ensure that pyrosequencing errors did not inflate the reported taxonomic richness, nor influence comparisons between communities. Because the vast majority of abundant tags had exact matches in the reference databases, the loss of taxonomic resolution in the current study would have affected mostly a subset of the rare taxa.

### Exploring the Rare Biosphere in the Distal Human Gut

**Figure 2:**
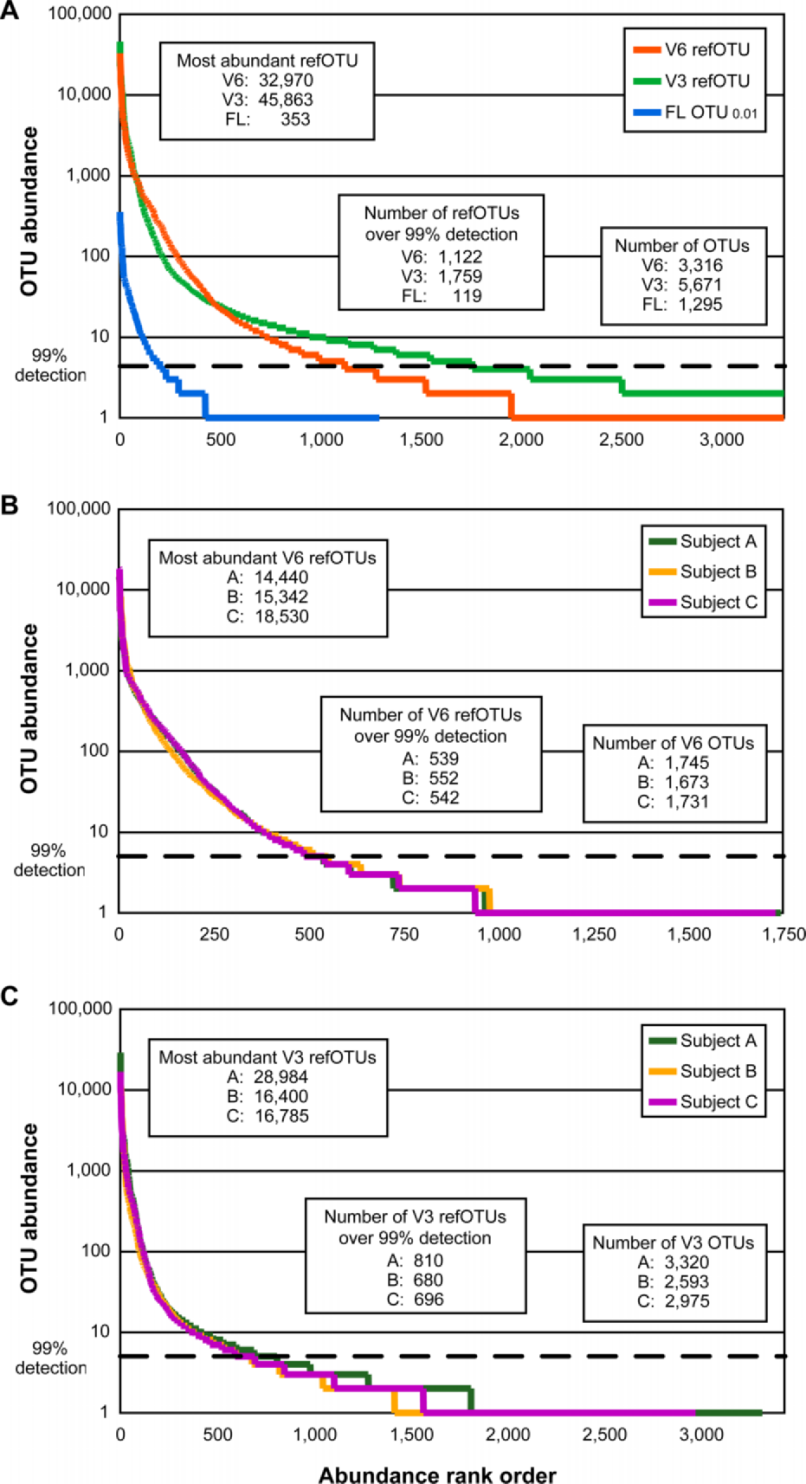
Rank Abundance Curves OTU abundance is shown on a logarithmic scale on the vertical axis; OTUs (defined as described in Methods) are listed in rank order along the horizontal axis. The dashed horizontal line at abundance 5 corresponds to an estimated probability of detection of 99%, based on the appearance of a rare OTU in a tag or sequence library according to the Poisson distribution. A comparison of V6, V3, and full-length libraries is shown in (A); the abundance per subject for V6 and V3 refOTUs are shown in (B and C). The maximum V3 refOTU abundance and number of V3 refOTUs are higher for individual A than other participants (C) because three additional samples were analyzed for this subject.

The rank abundance curves for OTUs derived from V6 and V3 tags and full-length sequences (Figure 2) show the extent to which tag pyrosequencing facilitated our exploration of the rare biosphere of the human gut. There was substantial agreement between the methods in the estimated relative abundance of specific taxa ranging from the phylum to genus level [48]. At a finer scale of resolution, the rank abundance curves had similar shapes for the V6 and V3 refOTUs and full-length OTU_0.01_s, with a small number of dominant taxa and a long tail of less abundant taxa (Figure 2A). Tag pyrosequencing identified many more OTUs than were identified by a large, traditional 16S rRNA survey, but of perhaps greater importance, there are many OTUs for which pyrosequencing provided a more precise estimate of relative abundance, and an improved confidence of detection. For example, 869 of the 1,295 OTU_0.01_s detected among the cloned full-length sequences were singletons, represented by only a single sequence. The best estimate for the relative abundance of each singleton OTU_0.01_ in the pooled community is 1/7,208 or 1.4 × 10^−4^, but the 95% confidence interval for this estimate (assuming a Poisson distribution of detection events) ranges over almost 2 orders of magnitude, 7.3 × 10^−6^–6.6 × 10^−4^. There is only a 63% chance for a specific taxon at 1.4 × 10^−4^ relative abundance to be detected in a single sample of 7,208 clones, so the presence (or absence) of these singleton OTUs does not provide a robust basis for comparisons between samples. A refOTU that appeared 65 times among the V6 or V3 tags would have had the same estimated relative abundance as a singleton OTU_0.01_ in the clone libraries, but with a much narrower 95% confidence interval of 1.0 × 10^−4^–1.7 × 10^−4^. There were 1,122 refOTUs in the pooled V6 libraries which occurred often enough (≥5 times) to estimate that their probability of detection was at least 99%; the estimated relative abundance for these taxa ranged from 7.4 × 10^−2^ to 1.1 × 10^−5^. The pooled V3 libraries had 1,759 refOTUs with at least 99% probability of detection, with relative abundance ranging over almost 4 orders of magnitude, from 9.3 × 10^−2^ to 1.0 × 10^−5^ Because the depth of pyrosequencing permits a high confidence of detection for many moderately rare and rare taxa, their presence or absence is informative for comparisons between samples. In contrast, in the pooled clone libraries, there were only 199 OTU_0.01_s (relative abundance ranging from 4.8 × 10^−2^ to 6.8 × 10^−4^), with at least a 99% estimated probability of detection.

Rank abundance curves for the V6 and V3 taxa within each individual are shown in Figure 2B and 2C. A range of 1,673–1,745 V6 refOTUs and 2,592–3,319 V3 refOTUs were found per individual, providing minimal estimates of the number of bacterial strains present in one person over an 8 month interval. Considering all the sampling times together, between 539–552 V6 refOTUs and 680–810 V3 refOTUs were found in each individual at 99% confidence of detection or above, compared to 66–98 full-length OTU_0.01_ per individual.

**Figure 3:**
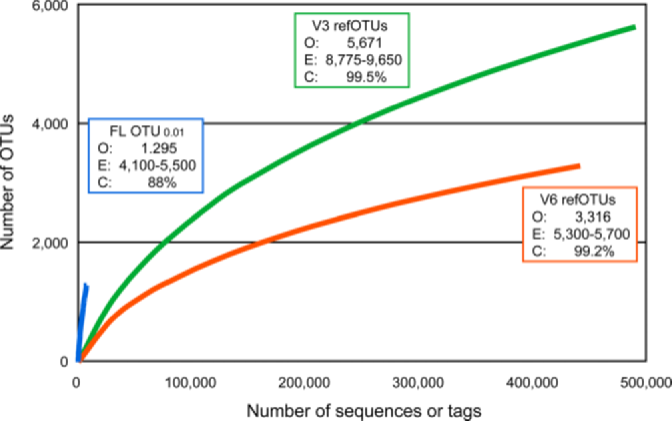
Rarefaction Curves The vertical axis shows the number of OTUs that would be expected to be found after sampling the number of tags or sequences shown on the horizontal axis. Curvature toward the horizontal indicates the increased sequencing effort required to observe novel OTUs when only rare OTUs remain to be discovered. Text boxes indicate the final observed OTU richness (O), range of estimated OTU richness by the ACE, ICE, Chao 1 and Chao 2 nonparametric estimators (E) [61–63], and Good's coverage (C) [64] for each method. The nonparametric estimators indicate that total OTU richness is much higher than currently observed, but are known to underestimate the true richness when the number of observations (i.e., tags or sequences) is small relative to the size of the community [60,63]. Good's coverage, the proportion of tags or sequences found in OTUs containing at least one other member, is an estimate of the probability that a tag or sequence drawn at random from the pool of amplicons will belong to an OTU that has already been detected.

Rarefaction curves can be used to assess the degree of completion of a taxonomic survey, i.e., how closely the observed taxonomic richness approaches the endpoint of all the taxa detectable with a particular method for that set of samples [60]. Figure 3 shows that the OTU_0.01_ rarefaction curve rises more steeply than the curves for V6 or V3 refOTUs, which reflects the greater taxonomic resolution of this typical OTU definition applied to full-length sequences, compared to the refOTU definitions for pyrosequencing tags. On the other hand, although this set of 7,208 sequences is among the largest datasets of full-length 16S rRNA sequences from the human microbiota (or any environment), the rarefaction curves for V6 and V3 tag pyrosequencing eventually rise higher and display more curvature toward the horizontal than the OTU_0.01_ curve. These features show that a single run of the FLX sequencer targeting V6 or V3 tags from the human gut microbiota can reveal more taxa, and capture a larger proportion of the detectable taxa, than a more extensive effort directed toward full-length 16S rRNA clone sequencing.

However, the tag-based surveys are by no means complete, as shown by the large number of singleton refOTUs in both the V6 and V3 data (1,361/3,316 and 2,306/5,671, respectively). Nonparametric estimators of total refOTU richness [61–63] were 60–70% higher than the observed richness, in the range of 5,300–5,700 refOTUs for the V6 tags and 8,875–9,650 refOTUs for the V3 tags. These large discrepancies between observed and estimated richness, and the 20–35% increase in estimated richness over the last half of sampling (Figure S1) indicate undersampling, a situation in which nonparametric estimators underestimate true richness [60,63]. The biological significance of the many rare, unobserved taxa implied by these estimates is unknown.

While rarefaction and richness estimators consider survey completion from the perspective of identified and unidentified taxa, coverage considers completion from the perspective of individual tags or sequences. Good's coverage [64], the estimated likelihood that a tag or sequence chosen from the sample at random will belong to an OTU that has already been identified in the dataset, was 99.2% and 99.5% for V6 and V3 data, respectively, in contrast to 88% for the full-length sequences. In other words, we expect that more than 100 additional tags would need to be sequenced in order to detect a new refOTU for either variable region, while on average less than ten new full-length sequences would suffice to detect a new OTU_0.01_.

### A Familiar Community with Fine-Scale Variation and Uncharacterized Taxa

**Figure 4:**
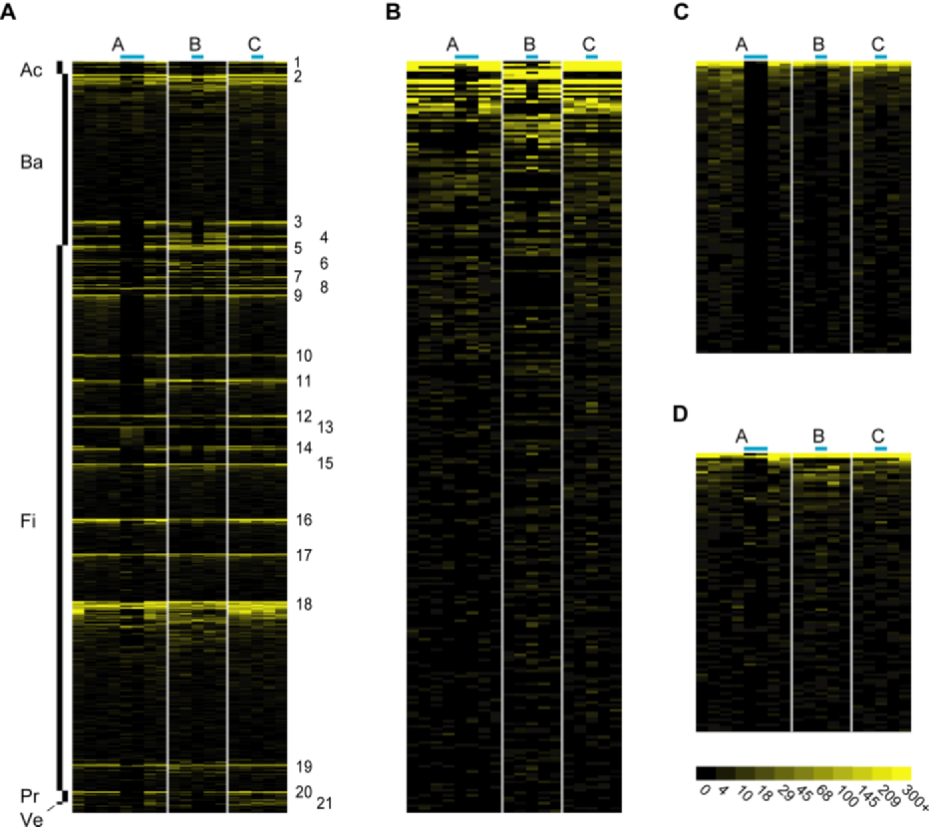
Relative Abundance of Taxa across Samples The heatmaps show the relative abundance per sample of V3 refOTUs with total abundance of at least ten, after normalizing to an equal number of tags per sample (see Methods). Color intensity for all panels is directly proportional to the logarithm of normalized OTU abundance from 0 to 300; the 473 highest refOTU abundance values per sample, ranging from 300 to 19,964, are shown at maximum color intensity. Each column in the heatmaps represents one sample, arranged chronologically from left to right within individuals; blue bars above columns indicate Cp-associated samples. Each row represents a V3 refOTU, arranged alphabetically in a taxonomic hierarchy from phylum to genus, and by decreasing abundance within a genus. (A) All 1,450 V3 refOTUs with total normalized abundance of at least ten. The staggered bars on the left indicate phyla (Ac = Actinobacteria, Ba = Bacteroidetes, Fi = Firmicutes, Pr = Proteobacteria, Ve = Verrucomicrobia). Numbers on the right represent prominent genera or higher taxonomic ranks when refOTUs could not be classified to the genus level (see Methods for details): 1. *Bifidobacteria*, 2*. Bacteroides*, 3. *Parabacteroides*, 4. *Alistipes*, 5*. Oscillospira*, 6. *Dialister*, 7*. Clostridium*, 8. *Dorea*, 9. *Faecalibacterium*, 10. *Subdoligranulum*, 11. *Clostridiaceae* (family), 12. *Eubacterium*, 13. *Anaerostipes*, 14. *Coprococcus* and *Lachnospira* (2 thin bands at this position), 15. *Roseburia*, 16. *Ruminococcus*, 17. *Lachnospiraceae* (family), 18. *Clostridiales* (order), 19. *Firmicutes* (phylum), 20. *Sutterella*, 21. *Akkermansia.* (B–D) The normalized relative abundance in detail (same data as (A)) for V3 refOTUs in the three most abundant genera, *Bacteroides, Faecalibacterium*, and *Roseburia*.

The heatmap in Figure 4A shows the relative abundance across 18 samples of 1,450 V3 refOTUs with normalized total abundance of at least ten. (Because normalization factors are as high as two for some samples, this corresponds to at least five observed tags, the threshold for 99% probability of detection.) All the statistical comparisons between samples that follow (e.g., PCA analysis and the identification of taxa that vary in response to Cp treatment) use the normalized abundance of these 1,450 taxa. Normalized V3 refOTU abundance within the three most abundant genera, *Bacteroides*, *Faecalibacter*, and *Roseburia*, are shown in greater detail in Figure 4B–4D, and the complete refOTU by sample abundance matrix is provided as Dataset S3. The most striking features of Figure 4 are consistent with our current understanding of the human distal gut microbiota [1,2,29,30]: (1) Most members of the community belong to a small number of genera within the Bacteroidetes and Firmicutes phyla, with members of Proteobacteria and Actinobacteria occurring much less frequently. (2) The diversity of genera and subgeneric taxa is greater in the Firmicutes than Bacteroidetes. (3) The same genera tend to be abundant or rare across all participants and all time points during unperturbed states, but at a finer taxonomic scale, the gut microbiota is individualized—some refOTUs are abundant in only one or two individuals.

Additional observations can be made from Figure 4 and Dataset S3 that are also consistent with previous data, although perhaps not as widely appreciated: (4) Temporal variability that is not associated with Cp treatment existed within each individual, (especially individual A) although it was not as evident as interindividual variation. (5) A total of ten phyla were found in the gut microbiota of these participants (six–nine per individual), some of which were discovered only recently. The fifth most abundant phylum was Verrucomicrobia (described in 1997) [65], within which almost all tags were associated with the genus *Akkermansia* (described in 2004) [66]. *Akkermansia* was of comparable abundance to the entire family Enterobacteriaceae including E. coli, the first described species of colonic bacteria*.* (6) In 2008, abundant, uncharacterized bacterial taxa are still found in the human gut. The sixth most abundant refOTU was V3_*Clostridiales*_refOTU_2, accounting for 3.5% of all V3 tags. The dominant tag in this refOTU exactly matched 203 cloned sequences in the V3 RefDB, but no sequences from cultivated organisms. Of these 203 sequences, 29 are shorter than 1,200 nucleotides or possibly chimeric; the remaining 174 sequences form a clade to the exclusion of any cultivated organisms. Pairwise genetic distances within this clade averaged 0.9%, while distances from sequences within the clade to the most similar sequences from cultivated strains averaged 10%, suggesting the presence of a novel species divergent from cultivated bacteria at roughly the family level (Dataset S4 and Figure S2).

### Community-Wide Consequences of Host Individuality and Cp Treatment

**Figure 5:**
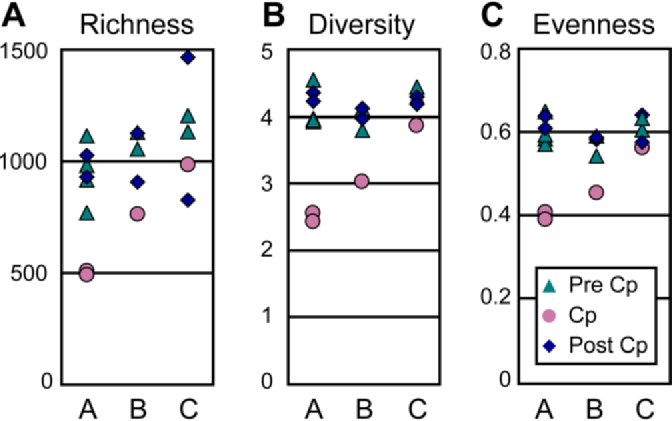
Diversity Statistics (A) Observed taxon richness (number of V3 refOTUs) per sample; Cp-associated samples have significantly fewer OTUs than pre- and post-Cp samples for individuals A and B (*p* < 0.005) but not individual C (*p* = 0.129). (B) Shannon diversity index; Cp-associated samples are significantly less diverse than other samples for all individuals (*p* < 0.001). (C) Shannon equitability index; OTU abundance in Cp-associated samples is significantly less evenly distributed than OTU abundance in other samples for all individuals (*p* < 0.001 for A and B, *p* < 0.05 for C). Formulas for diversity and evenness are given in Methods; significance is assessed as the probability that the Cp-associated value is drawn from the lower tail of a normal distribution with mean and variance as calculated from the other samples.

A dramatic effect of Cp on many taxa can also be seen in Figure 4, although the effect is apparently stronger in individuals A and B than individual C. Ecological diversity statistics confirmed this impression, as shown in Figure 5. Cp treatment significantly decreased taxonomic richness in the Cp-associated samples in individuals A and B (*p* < 10^−4^ and *p* < 0.005, respectively) but not C (*p* = 0.24). Cp treatment decreased the Shannon diversity index *H* (*p* < 10^−5^ in all cases) and Shannon equitability index *E_H_* (*p* < 10^−5^ for A and B, *p* < 0.05 for C) in all three individuals, although the magnitude of the effect was smaller for C. The magnitude of the Cp effect on diversity is best conveyed by transforming the Shannon index to an effective number of species [67,68], which is reduced 82%, 63%, and 36% in the Cp-associated samples of A, B, and C, respectively (Figure S3). None of the indices showed a detectable effect of Cp by 4 wk after treatment.

**Figure 6:**
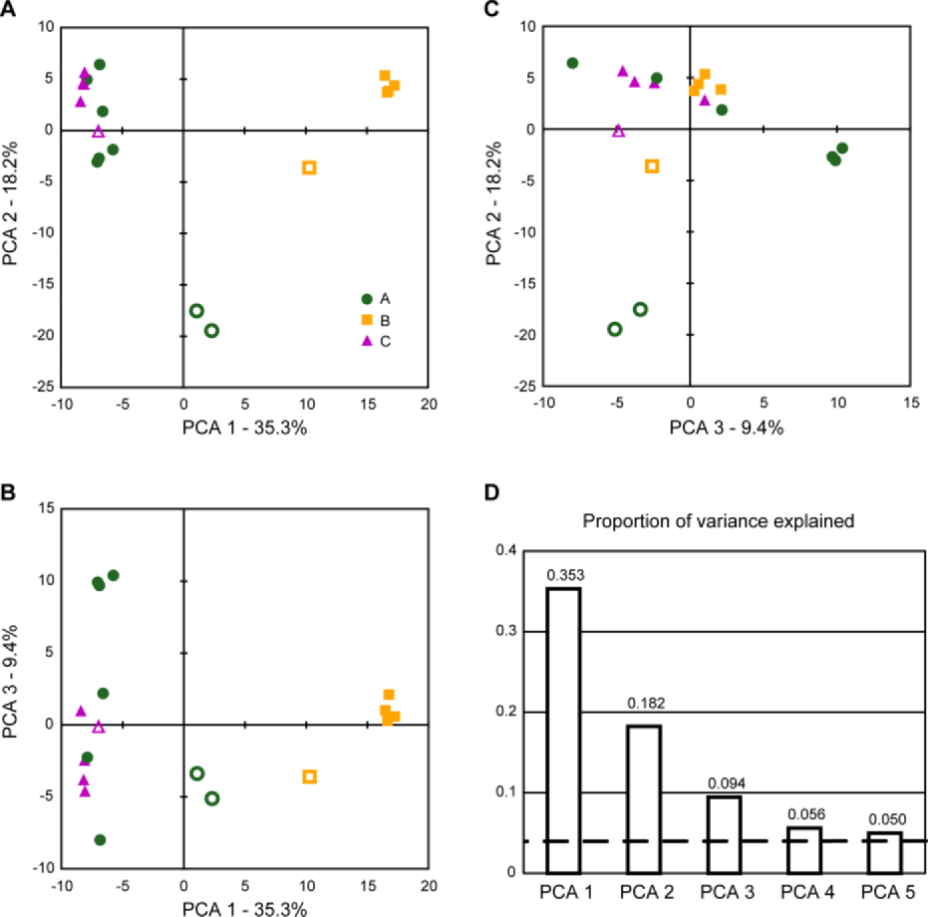
PCA of Relative Taxon Abundance Principal component analysis (PCA) of log_2_-transformed normalized abundance for 1,450 V3 refOTUs with normalized total abundance of at least ten (same data as in Figure 4A). Cp-associated samples are shown with open symbols, other samples with filled symbols. (A) PCA axis 1 (accounting for 35.3% of intersample variation) separates the samples of individual B from those of individuals A and C; PCA axis 2 (18.2% of intersample variation) separates Cp-associated samples from others. The separation of the Cp-associated samples from others is greatest for individual A, intermediate for B, and least for C, consistent with the heatmaps (Figure 4) and diversity statistics (Figure 5). (B) PCA axis 3 (9.7% of intersample variation) captures temporal variability in non-Cp samples of individual A, and to a lesser extent, individual C. Three samples collected from individual A in the week prior to Cp treatment cluster together at one extreme of the range of axis 3 values. (C) PCA axis 2 versus PCA axis 3. (D) Scree plot showing the proportion of variance explained by the first 5 PCA axes; the dashed line at 0.059 indicates the amount of variance expected for each axis with random, uncorrelated data.

Principal component analysis (PCA) of the log-transformed relative abundance of V3 refOTUs (Figure 6) confirmed that the primary sources of variability between samples were inter-individual differences and Cp treatment. Differences between individual B and the others were captured largely by the first PCA axis, which accounted for 35.8% of all variability between samples. The Cp-associated samples separated from non-Cp samples of the same individual primarily along PCA axis 2, which accounted for 18.6% of inter-sample variability. The observation that Cp-associated samples of A and B are closer to each other along axis 1 than the non-Cp samples indicated that some of the abundant taxa that differed between these individuals at other times became less abundant following Cp treatment. The third PCA axis (9.7% of variability) appeared to be driven primarily by Cp-independent temporal variability in individual A, with the three samples collected in the one-week interval prior to Cp treatment clustering at one extreme.

In the now-familiar context of interindividual variability in the human microbiota [30,69–71], the similarity of the gut communities in individuals A and C was unexpected. None of the study participants are related, nor do they live or work together, and individuals B and C would appear to be the most similar to each other based on host traits such as gender, ethnicity, and geographic origin (Table 1). Hence, other factors (e.g., diet) that were not investigated in this study may have an important influence on the similarity of the intestinal communities from individuals A and C.

Log transformation of taxon relative abundance prior to PCA reduces the influence of abundant taxa relative to that of moderately abundant taxa; it has an impact intermediate between performing no transformation, which allows abundant taxa to dominate inter-sample variability, and scaling the data so that the variability of each taxon among samples is weighted equally. Not surprisingly, the first axes of PCA on the untransformed refOTU data explained a higher proportion of the total variability (because a few taxa accounted for most of the variation); in this case, the distinction between the Cp-associated and non-Cp samples of individual C was obscured within the temporal variability of non-Cp samples of individual A (Figure S4A). On the other hand, with scaled PCA, the proportion of explained variability on the initial axes was lower, but the Cp-associated samples of both A and C were clearly separated in the same direction along axis 2 from a cluster containing all the non-Cp-associated samples from both individuals (Figure S4B). As with the diversity statistics, PCA (regardless of the abundance transformation) did not reveal any Cp effect at 4 wk post-treatment that could be distinguished from other sources of temporal variability. A caveat to interpreting PCA with any weighting scheme is that each refOTU is treated as equally dissimilar to all others. Preliminary results from ordination techniques that considered a range of refOTU relatedness showed a smaller magnitude of Cp-associated variability relative to the magnitude of interindividual differences of community composition (unpublished data).

### Specific Taxa Involved in Community Variation

**Figure 7:**
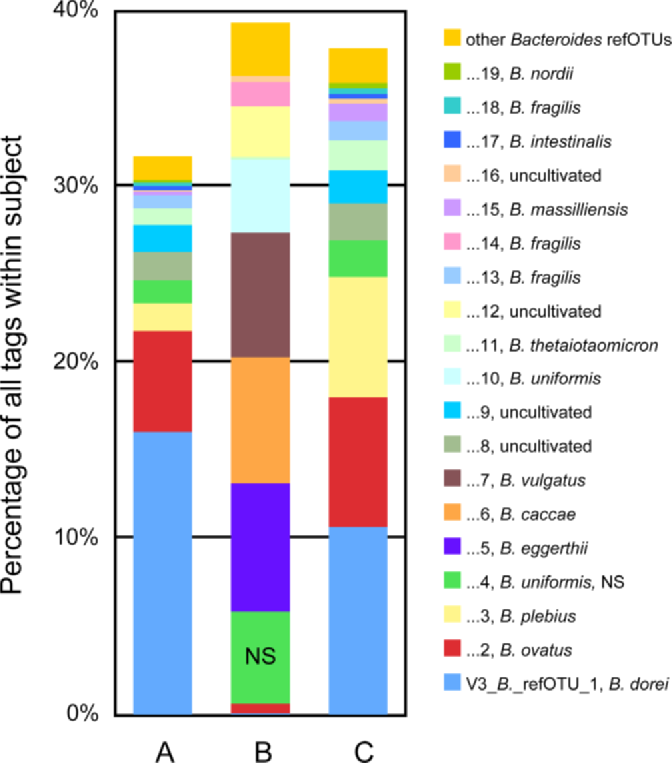
Abundant Taxa in the genus *Bacteroides* The relative abundance per subject of *Bacteroides* V3 refOTUs, expressed as the percentage of all V3 tags from that subject. The 19 most abundant *Bacteroides* refOTUs range from the first to the 91st most abundant taxa overall. Eighteen of these 19 *Bacteroides* refOTUs differ significantly in abundance between subjects (*p* < 0.05, FDR = 1.9%, NS marks the exception), but the abundance of the *Bacteroides* genus as a whole does not (*p* = 0.075, FDR 15%). Numbers in the legend indicate the rank order normalized abundance of refOTUs within *Bacteroides*, and correspond to the designation of the OTU according to the convention of this report (i.e., “1” represents V3_*Bacteroides_*refOTU_1.) The dominant tag in 15 of the 19 most abundant *Bacteroides* refOTUs has a perfect match to reference sequences from the cultivated species named in the legend.

Over a third of the V3 refOTUs with normalized total abundance of ten or more (528/1,450) differed significantly in abundance between participants (*p* < 0.05, 2.9% FDR). The taxa that varied between subjects included 64 of the 100 most abundant refOTUs, i.e., those representing 0.2% or more of all tags. An examination of these abundant taxa demonstrated the importance of maintaining high phylogenetic resolution during the analysis of tag pyrosequencing data (Dataset S5). The 64 abundant refOTUs that varied between subjects represent 23 different genera or other most-resolved taxa above the genus level. However, when these genera and more inclusive taxa were compared, only 11 of the 23 taxa were found to differ between individuals (*p* < 0.05). For example, 19 of the 100 most abundant V3 refOTUs belonged to the genus *Bacteroides*, and 18 of them differed significantly between individuals (Figure 7). However, the abundance of the *Bacteroides* genus as a whole did not differ significantly between individuals.

We tested taxa for a significant response to Cp by looking for two patterns: either a difference between the relative abundance in Cp-associated samples versus all other samples (pattern 1), or a difference between the relative abundance in pre-Cp samples versus samples collected during and after treatment (pattern 2). A detectable Cp response in a particular taxon could be a direct effect of Cp activity, or it could be mediated by ecological interactions with other taxa. Because these data represent relative abundance, a taxon that maintains constant abundance in absolute terms as the total community abundance declines will represent an increasing proportion of the community. For comparisons across all individuals we expressed the refOTU abundance in a sample as the deviance of log abundance from the mean log abundance of the refOTU in that individual; this procedure compared changes in relative, not absolute abundance across individuals. Cp effects were found in 30% of refOTUs (442/1,450) when tested over all individuals, more according to pattern 1 (428 taxa, *p* < 0.05, 7.7% FDR) and fewer according to pattern 2 (46 taxa, *p* < 0.005, 10% FDR; 32 taxa are significant according to both patterns) (Dataset S6). The predominance of pattern 1 was expected based on the PCA results shown in Figure 6; Cp-associated samples differed from others within each subject, but no separation of pre- and post-Cp samples was evident on the primary PCA axes.

**Figure 8:**
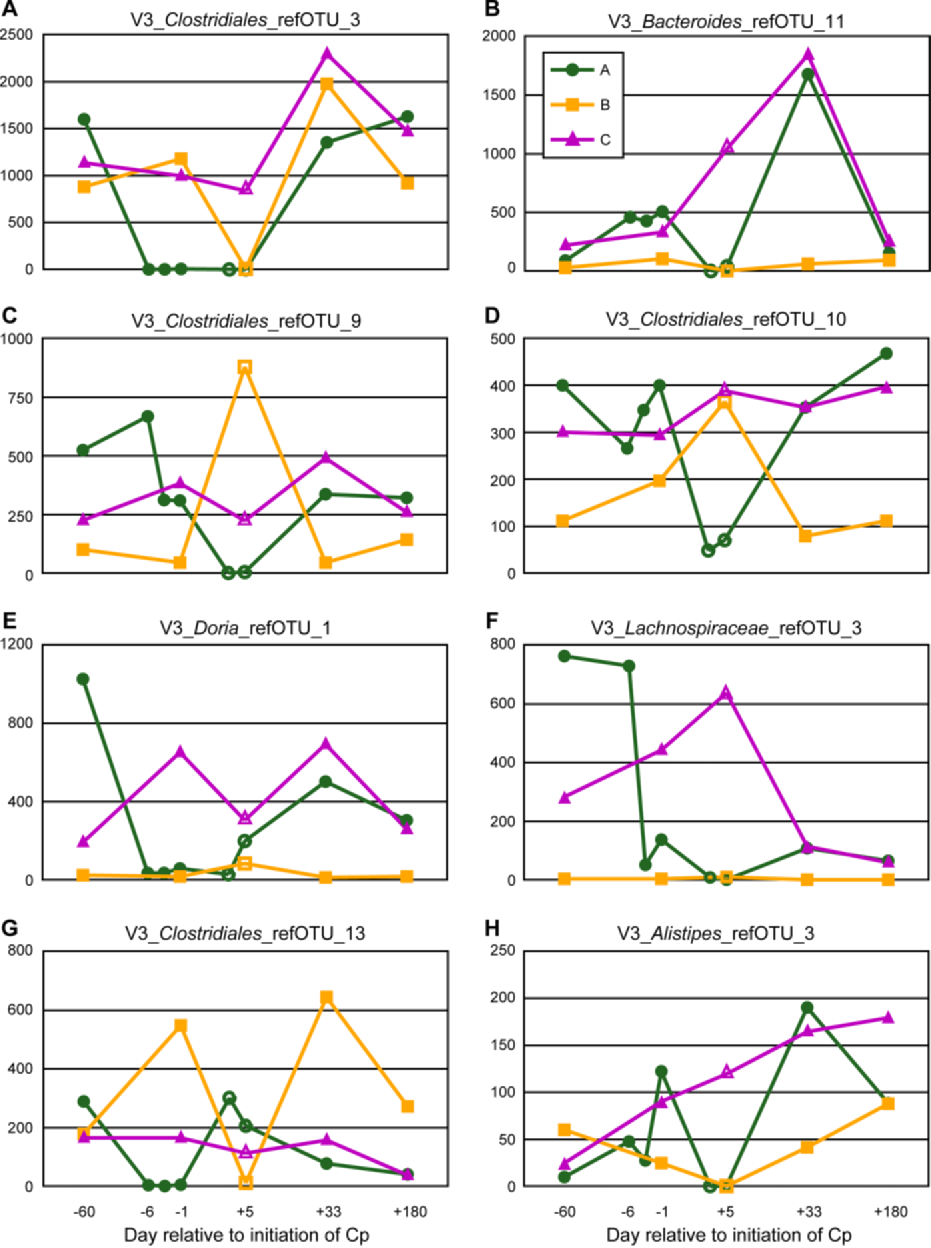
Individualized Cp Responses Normalized abundance is shown on the vertical axis for selected V3 refOTUs (scales differ between panels); samples are ordered chronologically along the horizontal axis. Cp-associated samples are shown with open symbols. (A–H) Eight taxa among the 90 most abundant V3 refOTUs that show contrasting responses to Cp among individuals. Temporal variability is also evident at times not associated with Cp use.

A number of the abundant taxa found to vary significantly in response to Cp appeared to have different responses among the participants (Figure 8), which suggested that testing for a Cp response within each individual might reveal additional taxa that respond to Cp only in some subjects. Testing only within A, for example, revealed that 25% (314/1,260) of refOTUs varied in response to Cp (pattern 1: 314 taxa significant, *p* < 0.05, 6.9% FDR; pattern 2: 1 taxon significant, *p* < 10^4^, 1.5% FDR, this taxon was significant by pattern 1 as well) (Dataset S7). Slightly less than half of these taxa (158/321) were found to be Cp responsive when testing across all individuals; the taxa found to be significant both within A and across all individuals were concentrated among the more abundant taxa. With data from only a single Cp-associated sample, we cannot yet conduct a similar comparison within individuals B and C, but the Cp response of the intestinal microbiota appears to be individualized, particularly for less abundant members of the community.

## Discussion

We applied the 16S rRNA tag pyrosequencing strategy of Sogin et al. [39] to characterize bacterial populations in the distal human gut, which constitute the predominant community of the human microbiota and one of the most densely populated microbial habitats on Earth. This approach revealed both the pervasive effects of an antibiotic that is considered to be relatively benign for the gut microbiota, and the resilience of the human gut microbiota following perturbation. As shown with marine, soil, and macaque gut samples [39,42,45], massively parallel pyrosequencing permits the exploration of microbial diversity in complex communities to an unprecedented depth. In comparison to clone libraries and traditional dideoxy sequencing, tag pyrosequencing detects more taxa, and provides more-accurate estimates of the relative abundance of a large number of moderate- and low-abundance taxa. Such data will facilitate better ecological studies of the human microbiota, with immediate clinical application. For example, the composition of the microbiota at the start of antibiotic treatment may determine the likelihood of pathogen overgrowth and life-threatening antibiotic-associated diarrhea (AAD) [72,73]; investigation of this hypothesis will depend on comprehensive characterizations of the community over time, including both rare and abundant taxa. Of the four bacterial pathogens associated with severe AAD, tags matching Klebsiella oxytoca and *Clostridium perfringens* were detected rarely in the current study (nine tags in two subjects and one tag, respectively); tags matching Clostridium difficile or Staphylococcus aureus were not found. The rarity of these pathogens and the absence of severe AAD in the current study is not surprising, since only a small number of healthy young adults were involved. It would be of tremendous interest and practical value to conduct a similar study of stool samples collected over time from elderly patients in hospitals or long-term care facilities, which have higher frequencies of both antibiotics use and serious complications from such use.

Cp is reported to have a lower rate of common gastrointestinal side effects than some other broad-spectrum antibiotics, and effects on gut microbial diversity (as detected by a low resolution “fingerprinting” technique) were less pronounced for Cp than for clindamycin or amoxicillin-clavulanic acid [25]. Nonetheless, the relative abundance levels of about 30% of the taxa in the gut were affected by Cp treatment when the comparison was made across all individuals, and additional taxa were found to respond to Cp within a single participant. Some of these changes may be direct effects of varying sensitivity to the antibiotic among the taxa comprising the gut microbiota. The refOTUs that increased in relative abundance following Cp treatment may represent taxa with intrinsic resistance to the antibiotic, strains that are typically Cp-sensitive but that had already acquired resistance prior to this study, or strains that developed Cp-resistance due to the current exposure. However, many of the changes in the community are likely to be explained by indirect effects, mediated by ecological interactions among taxa such as resource competition, cross-feeding, or the cooperative lysis of polymeric substrates [74,75].

Despite a pervasive disturbance of the gut microbiota, gut function remained normal as assessed subjectively by the participants, and the community composition in samples taken 4 wk after treatment were within the range of temporal variability of pretreatment samples. While the current study did not include samples showing the temporal features of the return of the community to its prior state, a pyrosequencing-based investigation of this rapid transition in a complex community is clearly of interest, and is continuing in our laboratory. The apparent continuity of gut function supports the hypothesis that the diversity of the microbial community provides functional redundancy [2]. At the same time, continuity in the predominant metabolic activity of the community, i.e., hydrolysis and fermentation of polysaccharides, does not necessarily imply the continuity of more specialized activities [7,76] such as bile transformation [77], immune modulation [9,78], or pathogen resistance due to specific inhibition [79,80]. Yet, the rapid return of the gut community to its pretreatment state in each individual suggests that not all communities supporting similar functions are equivalent. A quantitative examination of the metabolic transformations and host interactions of the gut microbiota during perturbation will be necessary to assess functional stability. In addition, it will be important to examine the microbiota at different sites within the intestinal tract in order to appreciate possible local antibiotic effects that are not revealed in fecal specimens. Epidemiological studies associating antibiotic-induced changes in the gut microbiota with chronic diseases [15,81] suggest that some consequences of community change may not be evident immediately.

An investigation into the factors responsible for community resilience is warranted. A mixture of selective forces intrinsic to the community (e.g., a competitive hierarchy based on relative growth rates and substrate affinity or interference mechanisms) and imposed by the environment (e.g., composition of the diet and of host-derived substrates) is likely to be involved, but nonselective forces such as re-colonization of the gut lumen from protected environments (perhaps the mucosa) must also be considered.

The current study can be compared to that of Young and Schmidt, who investigated changing bacterial populations in stool samples from a single patient with self-resolving AAD subsequent to amoxicillin-clavulanic acid treatment [82]. These investigators noted the complete absence of sequences from *Clostridium* cluster XIVa on the fourth day of antibiotic treatment (down from 20% on day 0) and a reduction from 33% to 15% of sequences affiliated with the genus *Faecalibacterium*; these two groups include the majority of butyrate-producing bacteria in the human gut [83]. (Butyrate is the preferred energy source of colonocytes.) In contrast, none of the participants in the current study experienced AAD. While *Faecalibacterium* refOTUs declined in all participants in response to Cp, each individual had other refOTUs with dominant tags exactly matching known butyrate-producing organisms that maintained or increased their abundance during Cp treatment (e.g., V3_*Roseburia*_refOTU_1 in individual B matches Butyrivibrio fibrisolvens and Roseburia intestinalis [84], V3_*Anaerostipes*_refOTU_1, in individuals A and C, matches the unnamed butyrate-producing strains SS2/1 and SSC/2 [85]).

Although the community as a whole showed a substantial return to the pre-Cp composition within 4 wk of the end of Cp treatment, there were examples of taxa that were affected by Cp and did not recover. V3_*Clostridiales*_refOTU_23 was the 41st and 52nd most abundant taxon before Cp treatment in individuals A and C, respectively, and present in all pre-Cp samples (means of 206 and 158 tags per sample); it was completely absent from all samples after the start of Cp treatment. This refOTU includes perfect matches to clones obtained from the human gut and from swine manure lagoons, but none to a cultivated bacterium. The most similar cultivated species are Clostridium piliforme and C. colinum, both zoonotic pathogens. V3_*Bilophila*_refOTU_1 was the 82nd and 79th most abundant pre-Cp refOTU in individuals A and C (means of 60 and 91 tags/sample); it was reduced to an average of less than two tags per sample after Cp treatment began. V3_*Bilophila*_refOTU_1 responded differently in individual B, in whom it was absent from the Cp-associated sample but subsequently rebounded to pre-Cp levels. The dominant tag of this refOTU exactly matches *Bilophila wadsworthia,* a common gut microbe that is frequently isolated in cases of appendicitis and from intestinal and extraintestinal abscesses [86]. The clinical significance of these persistent Cp-induced changes is unknown, but some gut bacteria are known to have important health effects despite being present at moderate or low abundance. For example, Oxalobacter formigenes is the most important known oxalate degrader in the human gut, an activity associated with a reduced risk for the development of calcium oxalate kidney stones [22]. O. formigenes is found at low abundance in most children, but is absent from many adults, which is generally attributed to its susceptibility to many antibiotics [22]. Tags matching O. formigenes were not detected in the current study.

Certain abundant taxa responded differently to Cp in different individuals, as shown in Figure 8. This result is not surprising when we consider that antibiotic resistance, even high-level Cp resistance resulting from chromosomal mutations [87], can be acquired far more rapidly than the rate of evolutionary change in the 16S rRNA gene. Although the participants had not taken any antibiotics in the year prior to the study, their earlier exposure to Cp or other antibiotics is unknown. Once established, antibiotic resistant strains can persist in the human gut for years in the absence of further antibiotic use [28]. Another potential explanation for divergent Cp responses of the same refOTU between individuals is the existence of indirect effects; the response may be mediated by ecological linkages that differ between individuals.

As shown in the companion paper of Huse et al. [48], the inferred bacterial community composition in these samples at taxonomic levels from phylum to genus was very similar, whether based on tags from the V6 or V3 variable regions or on full-length 16S rRNA sequences. All three techniques involve PCR amplification, and hence could fail to detect certain phylogenetic groups or lead to skewed levels of tag or sequence relative abundance with respect to the starting material [88,89]. However, the similarity of the results obtained from three distinct sets of primers suggests that the PCR bias of these primers (and the cloning bias for full-length sequences) for the bacterial taxa present in this habitat is minimal. The interpretation of these results as a measure of the relative abundance of cells in the gut habitat must still be made with caveats regarding differential cell lysis and the unequal numbers of rRNA genes per genome, as for any 16S rRNA-based technique [88,89].

Clearly, full-length sequences will provide the highest possible phylogenetic resolution for any genetic locus. However, pyrosequencing tags from either of two carefully chosen, short variable regions of the 16S rRNA gene have sufficient resolution to reveal taxonomic richness that exceeds any previously observed for samples of host-associated microbial communities, even though most diversity in these habitats is concentrated at the species and strain level [2,29]. This success occurred despite our use of reference databases to define OTUs, which necessarily constrained the resolution of tag pyrosequencing to the diversity already represented in public 16S rRNA sequence databases. A more accurate assessment of microbial diversity that accounts for the novel 16S rRNA variants discovered by pyrosequencing will depend on defining OTUs with reference to the tags themselves (e.g., using tag sequence divergence), with appropriate treatment of potential pyrosequencing errors. A high proportion of tags in the current study have an exact match in the database, due to the substantial sequencing effort that has already been directed to the human gut microbiota [7,30,90]. Hence, the constraint of defining OTUs with respect to a database is not as severe for this habitat as it may be for others [39,42]. However, the limitations of full-length 16S rRNA sequence surveys for discovering rare members of the gut biosphere are demonstrated by the contrast between the abundant tags in this study, almost all of which have an exact match in the database, and rare tags, which occur at a range of distances from their nearest database match (Figure 1).

This study has confirmed the existence of more than 5,600 bacterial taxa in the human gut, exceeding earlier predictions made on the basis of nonparametric richness estimators and full-length 16S rRNA sequencing studies [29,30], but the presence of singleton OTUs in our data leads to a still larger predicted richness for this habitat. As has long been appreciated, the highly uneven abundance distribution among taxa in this community (Figure 1, Dataset S3) hampers the identification of rare taxa via traditional sequencing approaches. However, coverage values exceeding 99% for our V6 and V3 data correspond to estimates that fewer than 1% of all detectable 16S rRNA tags in these samples belong to taxa that have not yet been detected. Individually, any of the rare, unobserved taxa in these samples are unlikely to exceed 0.001% of all 16S rRNA genes, the relative abundance at which we have roughly 99% confidence of detection. Because so many distinct reference tags are matched exactly by the tags obtained in this study, we conclude that a large number of bacterial taxa previously identified as gut inhabitants in a range of previous 16S rRNA sequencing studies can be present simultaneously or sequentially in a single individual, albeit at widely varying abundance. As suggested in a recent revision to the maxim of Baas Becking, “Everything may be everywhere, but not in equal amounts” [91]. This result challenges microbial ecologists to account for persistent differences in the composition of the microbiota between individuals, either by ongoing selective forces that vary among individuals such as diet and host genotype, or by factors such as founder effects and specific mutualistic interactions that stabilize community composition. However, any explanation will also have to account for the temporal variability shown by some taxa, and the resilience of the community to perturbations involving a significant fraction of its constituent populations.

## Supporting Information

Dataset S1 RefDB Hits for Abundant V6 and V3 Tags The file lists all the full-length sequences that contain the variable region tag that is the best RefDB hit (or that contain one of the set of equidistant best RefDB hits) to the 100 most abundant V6 and V3 tags.
(7.57 MB XLS)

Dataset S2 Assessment of Nondominant Tags in a refOTU as Pyrosequencing Error Products The file implements the G test to compare the sample distribution of the dominant tag in the 10 most abundant V6 and V3 refOTUs to the sample distribution of less-abundant tags in the same refOTU. By identifying significant differences in the sample distribution, the test can distinguish some genuine low-abundance tags from pyrosequencing error products.
(327 KB XLS)

Dataset S3 V6 and V3 refOTU Abundance Matrices The file gives the sample distribution and taxonomic classification of all V6 and V3 refOTUs.
(2.84 MB XLS)

Dataset S4 Genetic Distances between V3_*Clostridiales*_refOTU_2 and the Nearest Cultivated Strains The file contains a distance matrix including 174 full-length 16S rRNA sequences containing a V3 region that is identical to the dominant tag in V3_*Clostridiales*_refOTU_2, as well as 54 full-length 16S rRNA sequences from the most closely related cultivated strains.
(543 KB XLS)

Dataset S5 Comparison of V3 refOTU Abundance between Participants The file contains EDGE software output listing individual P values and cumulative FDRs (known as Q values) for a test of interindividual variation in V3 refOTU abundance.
(189 KB XLS)

Dataset S6 Assessment of a Cp Response in V3 refOTU Abundance across all Participants The file contains EDGE software output listing individual P values and cumulative FDRs (known as Q values) for tests of a Cp response in V3 refOTU abundance in all participants according to pattern 1 (Cp-associated versus other samples) and pattern 2 (pre-Cp versus post-Cp).
(251 KB XLS)

Dataset S7 Assessment of a Cp Response in V3 refOTU Abundance in Individual A The file contains EDGE software output listing individual P values and cumulative FDRs (known as Q values) for tests of a Cp response in V3 refOTU abundance in individual A according to pattern 1 (Cp-associated versus other samples) and pattern 2 (pre-ciprofloxacin versus post-Cp).
(219 KB XLS)

Figure S1 Rarefaction of OTU Richness Estimators Rarefaction curves of four nonparametric estimators of OTU richness, based on either the incidence (ICE, Chao 2) or relative abundance (ACE, Chao 1) of refOTUs in samples are compared to the observed refOTU richness for V6 (A) and V3 refOTUs (B). Both the large gap between observed and estimated OTU richness, and the upward trajectory of all estimators over the last half of sampling (right of the dashed line), indicate that the estimated richness is too low, and will continue to increase with additional sampling.
(772 KB TIF)

Figure S2 Uncultivated Taxon V3_*Clostridiales*_refOTU_2 The maximum-likelihood phylogenetic tree (calculated via AxML using default parameters in Arb [55]) shows the relationship between a clade containing 174 full-length V3 RefDB 16S rRNA sequences with exact matches to V3_*Clostridiales*_refOTU_2 and the most closely related sequences from cultivated strains. The tree was rooted using E. coli sequences as an outgroup (not shown). The strains shown in the figure were chosen as the closest neighbors to the V3_*Clostridiales*_refOTU_2 group based on a neighbor joining tree with 421 sequences comprised of all available type strain sequences from *Clostridiales* and *Lachnospirales*, and all full-length, high-quality sequences from cultivated strains identified by the RDP SeqMatch function [41] as one of the 20 nearest neighbors to any of eight V3 RefDB sequences chosen to represent the diversity within the V3_*Clostridiales*_refOTU_2 clade. Of 203 V3 RefDB sequences matching V3_*Clostridiales*_refOTU_2, 29 were excluded from the analysis as suspected chimeras due to one or more of the following: failure to align with the RDP automated aligner, being marked as “suspect: in the RDP database, or being flagged by Mallard [92] as a likely chimera in comparison to the set of sequences used in the neighbor joining tree above.
(1.44 MB TIF)

Figure S3 Cp Effects Assessed via Effective Number of Taxa Cp decreases the effective number of refOTUs [67] in the gut bacterial community as assessed by diversity of order zero (^0^D) (A), order one (^1^D) (B), and order two (^2^D) (C). Diversities of different orders vary in the relative importance of rare and abundant taxa in calculating a diversity index. The use of an effective number of taxa as the unit of diversity overcomes difficulties with traditional measures such as the Shannon-Weiner index (Figure 5B) and the Gini-Simpson index, which are measured in units that are not commensurate with each other, and that have no natural interpretation. An effective number of taxa is interpreted as the number of equally abundant taxa that would result in the same calculated diversity as the community under investigation. ^0^D disregards abundance to consider rare and abundant taxa equally important, thus it is equivalent to taxon richness as shown in Figure 5A. ^1^D considers that taxa contribute to diversity in proportion to their relative abundance (like the Shannon-Weiner index, compare Figures 5B and S3B), and ^2^D weighs taxa in proportion to the square of relative abundance (like the Gini-Simpson index). Cp-associated samples are significantly lower in ^1^D and ^2^D than other samples for all individuals.
(846 KB TIF)

Figure S4 Untransformed and Scaled PCA The transformation applied to taxon abundance data influences the similarity of samples as assessed by PCA. PCA on untransformed V3 refOTU abundance data emphasizes the small number of highly abundant taxa (A). In this case, the first PCA axis captures both community differences between individual B and the others, and differences between the communities of Cp-affected samples and other samples of individual A. The second PCA axis reflects differences in the Cp-associated samples of both individuals A and B. Changes of community composition in the Cp-associated sample of individual C were less distinct than in the other individuals (Figures 4–6), and with untransformed abundance data the Cp-associated sample of individual C falls within the cluster of non-Cp samples of individuals A and C. The influence of abundance can be removed entirely by scaling the data so that the inter-sample variance of each taxon is weighted equally (B). In this case the Cp-associated samples of both individuals A and C are displaced in the same direction along PCA axis 2 from the cluster of non-Cp samples of these individuals. However, the Cp-associated sample is less distant from the non-Cp samples within individual B. Considered together, these results suggest that the effects of Cp in individual C involve changes in the identity of less abundant taxa, while in individual B the effects also involve large changes in the abundance of taxa. Cp-associated changes in individual A involve many of the same taxa as in individual C, but changes in the abundant taxa are more pronounced.
(853 KB TIF)

Text S1 Supporting Methods Supporting methods for chimera identification, analysis of pyrosequencing errors, and rare genuine tags, and comparisons of taxon abundance across samples using a FDR criterion
(73KB XLS).

## Abbreviations

AAD: antibiotic-associated diarrhea
Cp: ciprofloxacin
FDR: false discovery rate
OTU: operational taxonomic unit
PCA: principal component analysis
