# Supplementary figures and images for: The Pervasive Effects of an Antibiotic on the Human Gut Microbiota, as Revealed by Deep 16S rRNA Sequencing
Source: PLoS Biol. 2008 Nov 18;6(11):e280. doi: 10.1371/journal.pbio.0060280 (PMC2586385; doi:10.1371/journal.pbio.0060280)

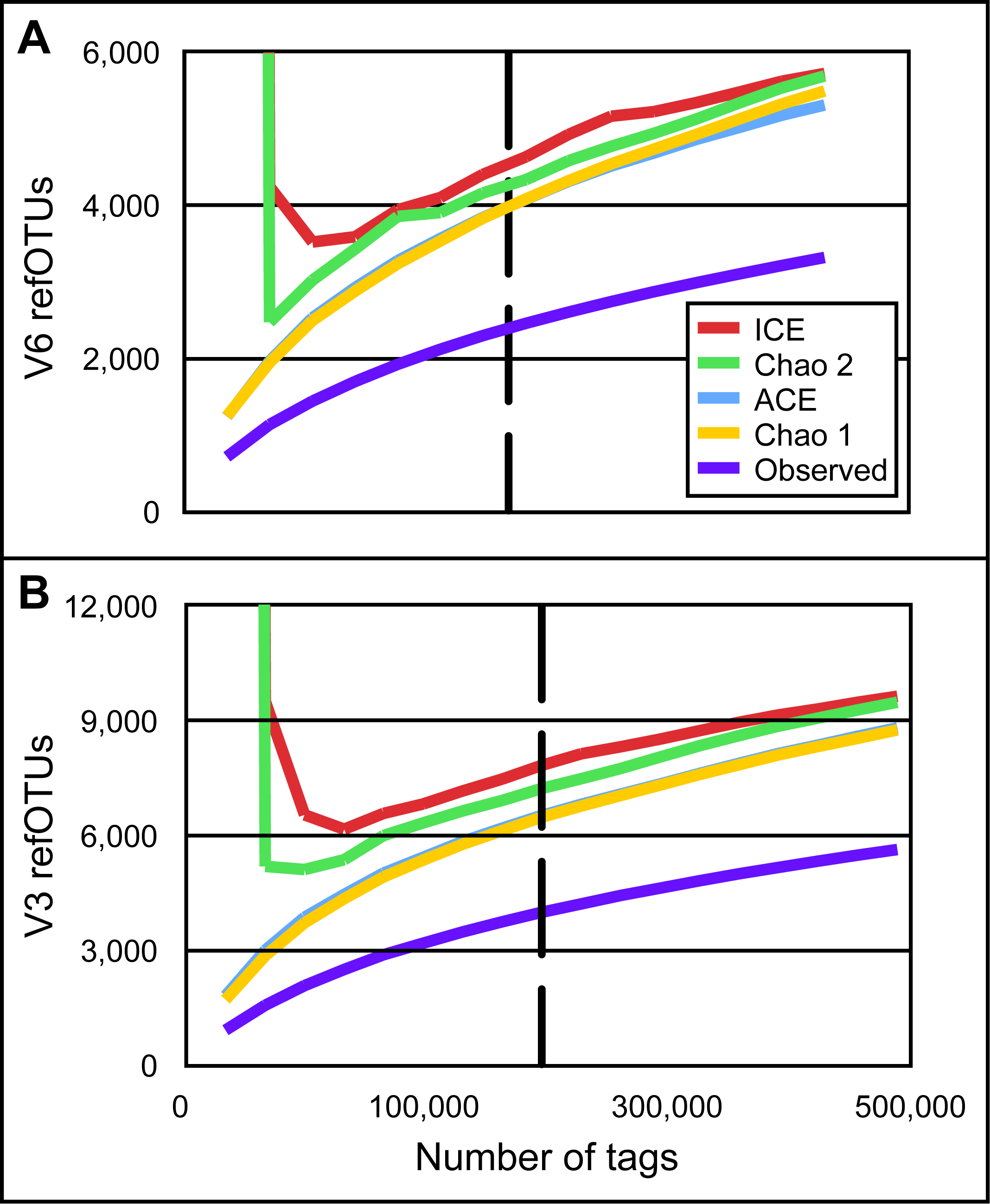

Supplement: Figure S1 — Rarefaction curves of four nonparametric estimators of OTU richness, based on either the incidence (ICE, Chao 2) or relative abundance (ACE, Chao 1) of refOTUs in samples are compared to the observed refOTU richness for V6 (A) and V3 refOTUs (B). Both the large gap between observed and estimated OTU richness, and the upward trajectory of all estimators over the last half of sampling (right of the dashed line), indicate that the estimated richness is too low, and will continue to increase with additional sampling. (772 KB TIF) [file pbio.0060280.sg001.tif]

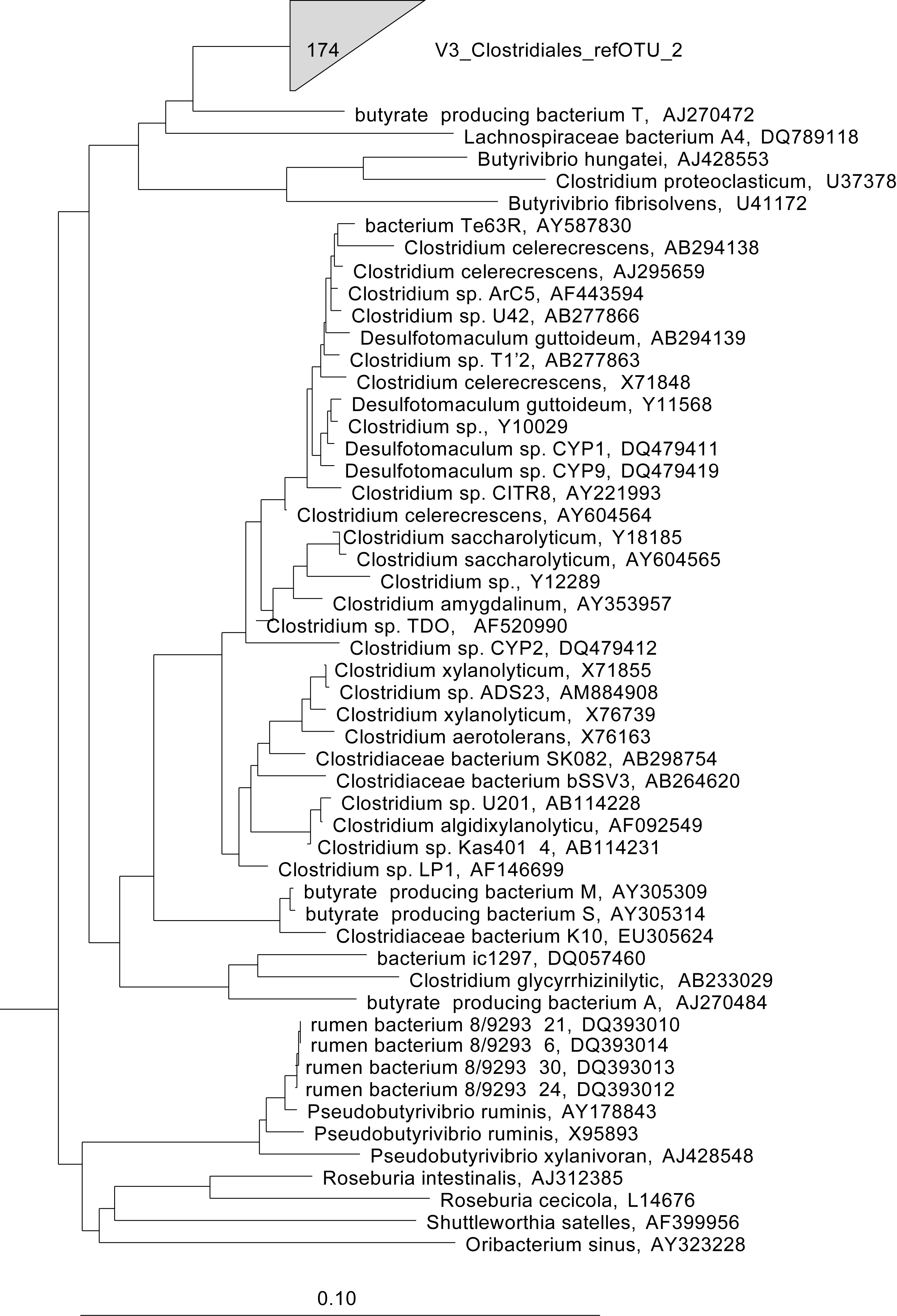

Supplement: Figure S2 — The maximum-likelihood phylogenetic tree (calculated via AxML using default parameters in Arb [55]) shows the relationship between a clade containing 174 full-length V3 RefDB 16S rRNA sequences with exact matches to V3_Clostridiales_refOTU_2 and the most closely related sequences from cultivated strains. The tree was rooted using E. coli sequences as an outgroup (not shown). The strains shown in the figure were chosen as the closest neighbors to the V3_Clostridiales_refOTU_2 group based on a neighbor joining tree with 421 sequences comprised of all available type strain sequences from Clostridiales and Lachnospirales, and all full-length, high-quality sequences from cultivated strains identified by the RDP SeqMatch function [41] as one of the 20 nearest neighbors to any of eight V3 RefDB sequences chosen to represent the diversity within the V3_Clostridiales_refOTU_2 clade. Of 203 V3 RefDB sequences matching V3_Clostridiales_refOTU_2, 29 were excluded from the analysis as suspected chimeras due to one or more of the following: failure to align with the RDP automated aligner, being marked as “suspect: in the RDP database, or being flagged by Mallard [92] as a likely chimera in comparison to the set of sequences used in the neighbor joining tree above. (1.44 MB TIF) [file pbio.0060280.sg002.tif]

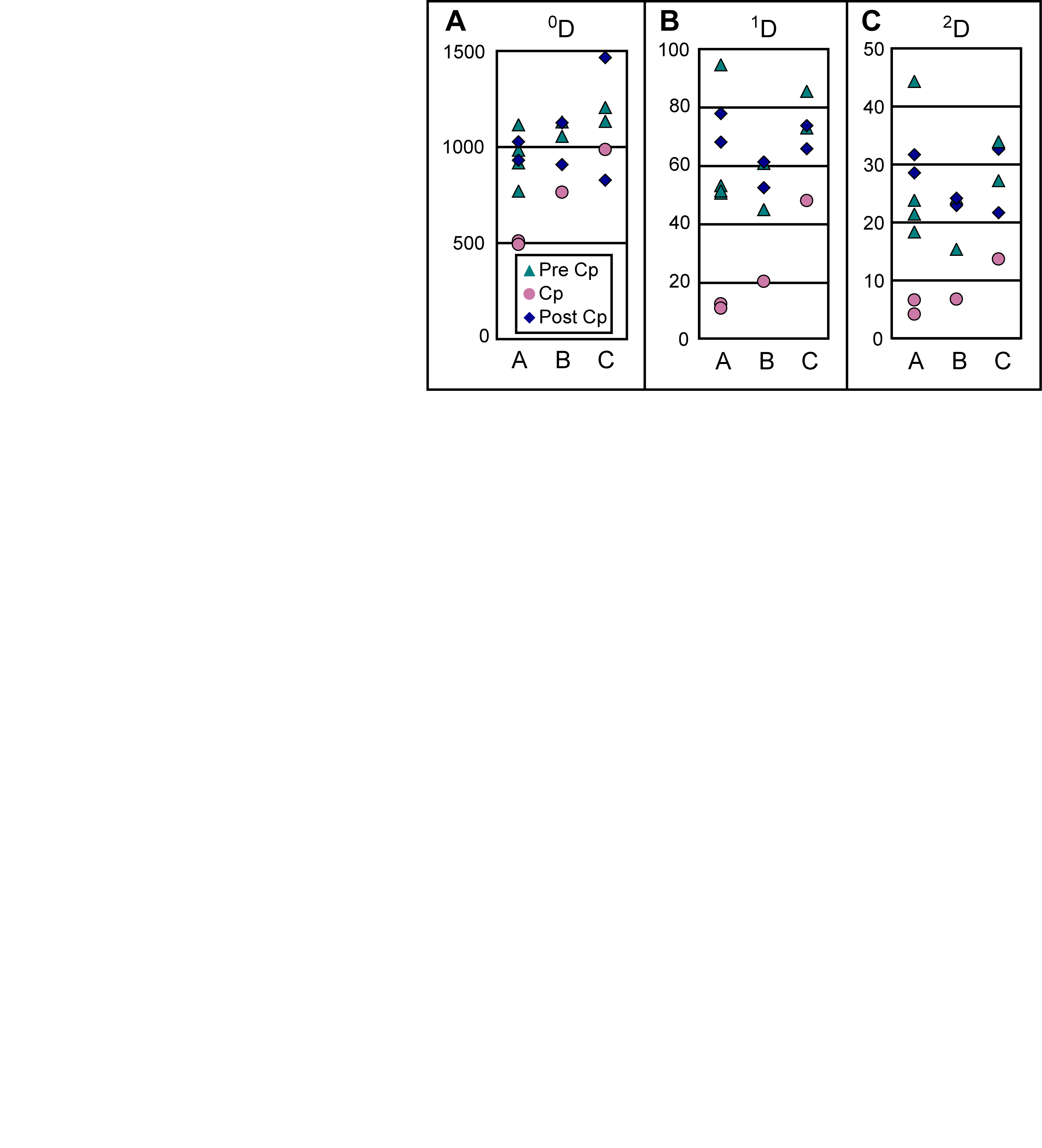

Supplement: Figure S3 — Cp decreases the effective number of refOTUs [67] in the gut bacterial community as assessed by diversity of order zero (0D) (A), order one (1D) (B), and order two (2D) (C). Diversities of different orders vary in the relative importance of rare and abundant taxa in calculating a diversity index. The use of an effective number of taxa as the unit of diversity overcomes difficulties with traditional measures such as the Shannon-Weiner index (Figure 5B) and the Gini-Simpson index, which are measured in units that are not commensurate with each other, and that have no natural interpretation. An effective number of taxa is interpreted as the number of equally abundant taxa that would result in the same calculated diversity as the community under investigation. 0D disregards abundance to consider rare and abundant taxa equally important, thus it is equivalent to taxon richness as shown in Figure 5A. 1D considers that taxa contribute to diversity in proportion to their relative abundance (like the Shannon-Weiner index, compare Figures 5B and S3B), and 2D weighs taxa in proportion to the square of relative abundance (like the Gini-Simpson index). Cp-associated samples are significantly lower in 1D and 2D than other samples for all individuals. (846 KB TIF) [file pbio.0060280.sg003.tif]

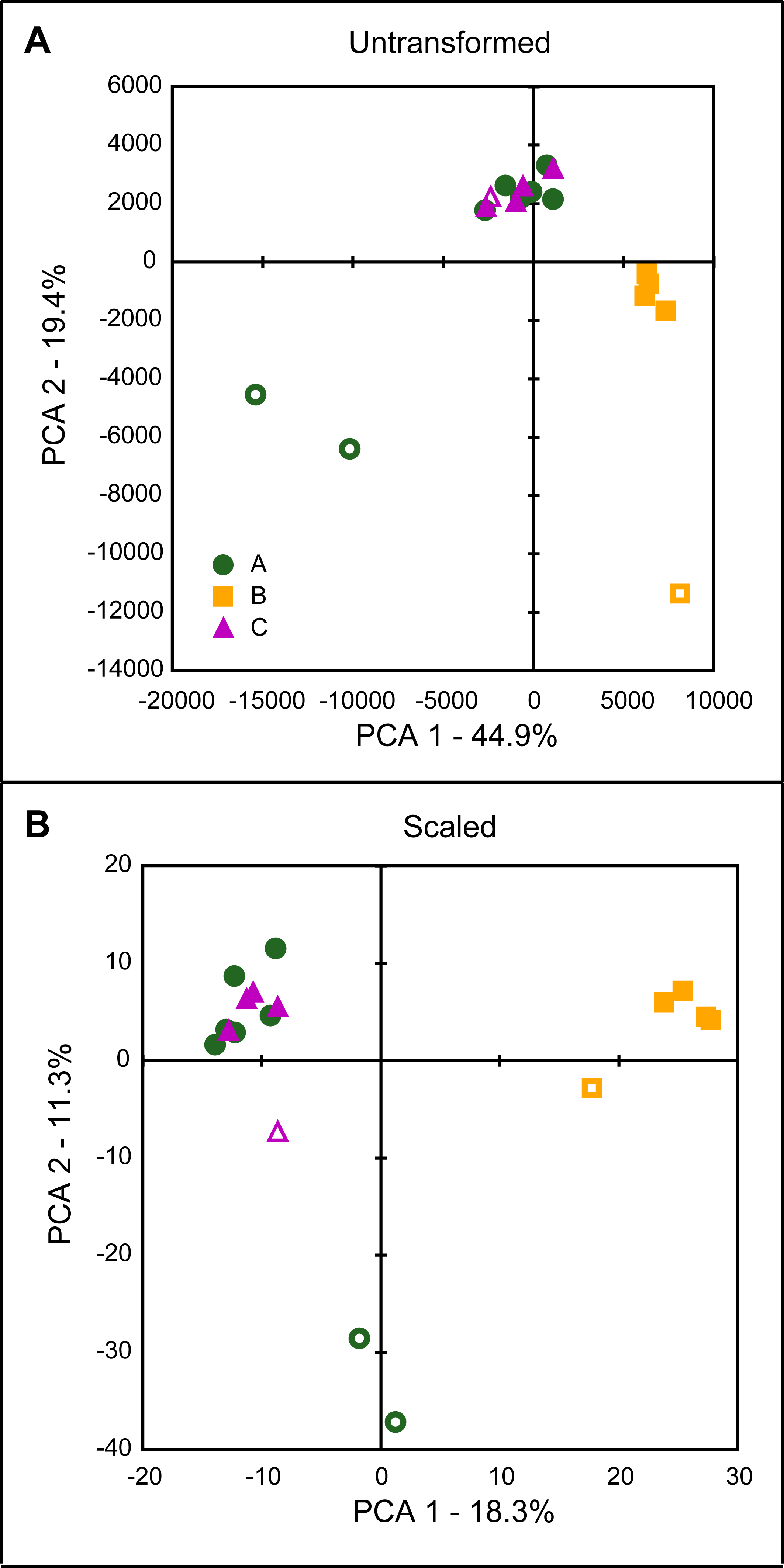

Supplement: Figure S4 — The transformation applied to taxon abundance data influences the similarity of samples as assessed by PCA. PCA on untransformed V3 refOTU abundance data emphasizes the small number of highly abundant taxa (A). In this case, the first PCA axis captures both community differences between individual B and the others, and differences between the communities of Cp-affected samples and other samples of individual A. The second PCA axis reflects differences in the Cp-associated samples of both individuals A and B. Changes of community composition in the Cp-associated sample of individual C were less distinct than in the other individuals (Figures 4–6), and with untransformed abundance data the Cp-associated sample of individual C falls within the cluster of non-Cp samples of individuals A and C. The influence of abundance can be removed entirely by scaling the data so that the inter-sample variance of each taxon is weighted equally (B). In this case the Cp-associated samples of both individuals A and C are displaced in the same direction along PCA axis 2 from the cluster of non-Cp samples of these individuals. However, the Cp-associated sample is less distant from the non-Cp samples within individual B. Considered together, these results suggest that the effects of Cp in individual C involve changes in the identity of less abundant taxa, while in individual B the effects also involve large changes in the abundance of taxa. Cp-associated changes in individual A involve many of the same taxa as in individual C, but changes in the abundant taxa are more pronounced. (853 KB TIF) [file pbio.0060280.sg004.tif]
