# Supplementary material for: The Pervasive Effects of an Antibiotic on the Human Gut Microbiota, as Revealed by Deep 16S rRNA Sequencing
Source: PLoS Biol. 2008 Nov 18;6(11):e280. doi: 10.1371/journal.pbio.0060280 (PMC2586385; doi:10.1371/journal.pbio.0060280)
Supplement: Text S1 — Supporting methods for chimera identification, analysis of pyrosequencing errors, and rare genuine tags, and comparisons of taxon abundance across samples using a FDR criterion (73KB XLS). [file pbio.0060280.sd008.doc]

**Text S1. Supporting Methods**

*Chimera identification*

Chimeric gene sequences result from molecular artifacts generated during PCR, wherein two (or more) gene fragments from distinct parental sequences are combined into a single, apparently complete gene sequence. DeSantis introduced the divergence ratio (DR) as the basis for chimera determination in the Greengenes implementation [1] of the Bellerophon chimera checking algorithm [2], with DR > 1.1 interpreted as evidence of a chimera by default. The DR numerator is the average sequence identity between the two identified fragments of a possible chimera and their putative parental sequences, while the DR denominator is the average sequence identity between the two putative parents. Because the numerator is at most 100%, DR cannot exceed 1.1 and a chimera determination cannot be made with the default settings if the two putative parental sequences are more than 90.9% identical. Examination of full length sequences in our dataset for which DR < 1.1, using Mallard, an alternative chimera detection algorithm [3], suggests that at least 10% of them are chimeric (data not shown). Furthermore, the Bellerophon algorithm assumes that chimeras have only two distinct fragments; both published results [3] and our own experience indicate that more complex chimeras are common, which has the effect of reducing the DR. Hence, we chose to exclude all sequences for which the Bellerophon algorithm identified two putative parental sequences, regardless of the DR.

To assess whether this stringent approach for chimera removal could have inadvertently influenced the inferred community composition, we used the LibCompare tool of the RDP website (<http://rdp.cme.msu.edu/>) [4] to compare the set of sequences we analyzed to the set of sequences retained by the default Bellerophon settings. Separate comparisons were done for each of the three subjects because LibCompare is currently limited to 5,000 sequences per library, precluding the analysis of all sequences in a single comparison. LibCompare, which compares the relative abundance between two sequence libraries of all taxa at the genus and more inclusive levels, found no taxa that differed significantly in relative abundance between the two sets of sequences, even without any correction for multiple tests (p > 0.1 in all cases).

*Analysis of pyrosequencing* *errors and rare genuine tags*

Abundant V6 and V3 refOTUs were almost always comprised of a single dominant tag with a perfect match to the RefDB (Figure 1) and many more tags of much lower abundance, most of which differed from the dominant tag at only one or two nucleotide positions. (Over the 100 most abundant refOTUs, the dominant tag comprised 92 ± 8% (mean ± sd) of the refOTU for the V6 region, and 85 ± 7% for the V3 region.) The dominant tag is presumably error-free, but the nondominant tags could be genuine biological sequences or pyrosequencing error products related to the dominant tag. We sought to identify genuine sequences among the nondominant tags belonging to the 10 most abundant V6 and V3 refOTUs using the following strategy: The likelihood of a single specific error (e.g., insertion of a G after position 20) occurring during pyrosequencing of a given amplicon is dependent on the amplicon sequence (e.g., the presence of GGGG in positions 17-20), but independent of the sample from which the amplicon was generated. Therefore, the expected distribution among samples of a single, specific pyrosequencing error product (e.g., tags differing from the error-free tag only by an additional G after position 20) is a fixed proportion of the distribution of the error-free tag from which it was derived. Taking the abundance ratio of a specific nondominant tag to the dominant tag across all samples as the expected ratio for each individual sample, we implemented the G test with the Williams correction [5] in spreadsheet software to look for significant deviation from this expectation for nondominant tags with total abundance across all samples of at least 25 (File S2). The minimum abundance criterion was necessary because the G test (like similar tests) lacks statistical power when the expected number of tags per sample is low. In addition, samples were pooled within subjects starting with samples of the same type (e.g., pre- or post-Cp) if the expected number of the nondominant tag in a sample (or pooled samples) was lower than 4.5. In some cases, all samples from a subject were pooled, but samples were never pooled across subjects.

*Comparisons of taxon abundance across samples using a false discovery rate criterion*

We used Edge software [6] for testing the significance of taxon abundance variation among samples, because of its implementation of an optimal false discover rate (FDR) procedure for controlling the rate of type I errors in a large number of independent tests [7]. Traditional methods of addressing the multiple testing issue (e.g., the Bonferoni correction) control the likelihood of a single type I error for all tests together; with tests on 1,484 V3 refOTUs, this approach would result in a tremendous loss of power (the ability to detect true positive results). Instead, in addition to requiring at least 95% confidence (p < 0.05) by permutation testing to declare that any individual taxon varied in relative abundance between conditions, we also imposed a maximum 10% expected false discovery rate (FDR). Either the individual p value or the comparison-wide FDR criterion may be more restrictive for a given comparison.

Abundance data for each refOTU were log-transformed after adding a random constant uniformly distributed between 0.5 and 1.5, and the mean log abundance was subtracted from the log abundance of each sample; we used the mean within individuals for Cp comparisons between individuals, and used the mean over all data in other comparisons. We centered the log abundance values around the mean log abundance in each individual for the inter-individual comparisons so the same relative change in refOTU abundance would be weighted equally in all of them, despite inter-individual differences in absolute abundance. We added a random constant centered at 1 (instead of adding 1, the typical procedure for log transformation) because permutation-based significance testing in the Edge program (designed for continuous-valued microarray fluorescence measurements, not discrete abundance data) depends on the abundance of all refOTUs having unequal variance across samples. This requirement would otherwise be violated for low abundance taxa that happened to share identical sample distributions. The random constant also has an effect similar to a continuity correction as recommended for count data [5], and reduces the chance of falsely declaring variation in low abundance taxa to be significant. We tested refOTUs for a response to Cp according to two different patterns, either that the abundance in Cp-associated samples differed from all others (pattern 1), or that the pre-Cp abundance differed from Cp-associated and post-Cp abundance (pattern 2). For each comparison, the list of taxa with significant variation was generated in order of ascending p values, until the next taxon had p > 0.05, or would result in an FDR exceeding 10% for the entire list.

1. DeSantis TZ, Hugenholtz P, Larsen N, Rojas M, Brodie EL, et al. (2006) Greengenes, a chimera-checked 16S rRNA gene database and workbench compatible with ARB. Appl Environ Microbiol 72: 5069-5072.

2. Huber T, Faulkner G, Hugenholtz P (2004) Bellerophon: a program to detect chimeric sequences in multiple sequence alignments. Bioinformatics 20: 2317-2319.

3. Ashelford KE, Chuzhanova NA, Fry JC, Jones AJ, Weightman AJ (2006) New screening software shows that most recent large 16S rRNA gene clone libraries contain chimeras. Appl Environ Microbiol 72: 5734-5741.

4. Wang Q, Garrity GM, Tiedje JM, Cole JR (2007) Naive Bayesian classifier for rapid assignment of rRNA sequences into the new bacterial taxonomy. Appl Environ Microbiol 73: 5261-5267.

5. Sokal RR, Rohlf FJ (1995) Biometry. New York: W.H. Freeman and Company. 887 p.

6. Leek JT, Monsen E, Dabney AR, Storey JD (2006) EDGE: extraction and analysis of differential gene expression. Bioinformatics 22: 507-508.

7. Storey JD, Dai JY, Leek JT (2007) The optimal discovery procedure for large-scale significance testing, with applications to comparative microarray experiments. Biostatistics 8: 414-432.
